# His domain protein tyrosine phosphatase and Rabaptin-5 couple endo-lysosomal sorting of EGFR with endosomal maturation

**DOI:** 10.1242/jcs.259192

**Published:** 2021-11-04

**Authors:** Gabrielle Parkinson, Peristera Roboti, Ling Zhang, Sandra Taylor, Philip Woodman

**Affiliations:** Faculty of Biology, Medicine and Health, Manchester Academic and Health Science Centre, The University of Manchester, Manchester M13 9PT, UK

**Keywords:** HD-PTP, PTPN23, Rabaptin-5, ESCRT, Multivesicular body, Rab5, Rabex-5

## Abstract

His domain protein tyrosine phosphatase (HD-PTP; also known as PTPN23) collaborates with endosomal sorting complexes required for transport (ESCRTs) to sort endosomal cargo into intralumenal vesicles, forming the multivesicular body (MVB). Completion of MVB sorting is accompanied by maturation of the endosome into a late endosome, an event that requires inactivation of the early endosomal GTPase Rab5 (herein referring to generically to all isoforms). Here, we show that HD-PTP links ESCRT function with endosomal maturation. HD-PTP depletion prevents MVB sorting, while also blocking cargo from exiting Rab5-rich endosomes. HD-PTP-depleted cells contain hyperphosphorylated Rabaptin-5 (also known as RABEP1), a cofactor for the Rab5 guanine nucleotide exchange factor Rabex-5 (also known as RABGEF1), although HD-PTP is unlikely to directly dephosphorylate Rabaptin-5. In addition, HD-PTP-depleted cells exhibit Rabaptin-5-dependent hyperactivation of Rab5. HD-PTP binds directly to Rabaptin-5, between its Rabex-5- and Rab5-binding domains. This binding reaction involves the ESCRT-0/ESCRT-III binding site in HD-PTP, which is competed for by an ESCRT-III peptide. Jointly, these findings indicate that HD-PTP may alternatively scaffold ESCRTs and modulate Rabex-5–Rabaptin-5 activity, thereby helping to coordinate the completion of MVB sorting with endosomal maturation.

## Abstract (precis)

**Summary:** Sorting of endocytic cargo to the multivesicular body is accompanied by endosomal maturation. Here, we provide a potential mechanism by which these two processes are linked.

## INTRODUCTION

Plasma membrane proteins that are internalised by endocytosis enter the early endosome and are then subject to a critical decision. Some proteins recycle to the surface, but those destined for degradation are ubiquitylated and sorted into intralumenal vesicles (ILVs) of the developing multivesicular body (MVB), en route to the lysosome (Naslavsky and Caplan, 2018; Norris and Grant, 2020; Weeratunga et al., 2020). The epidermal growth factor receptor (EGFR) is a clinically important example of an MVB cargo (Felder et al., 1990). Within the early endosome, ubiquitylated EGFR (Galcheva-Gargova et al., 1995) engages the endosomal sorting complexes required for transport (ESCRT) pathway (Eden et al., 2012; Raiborg and Stenmark, 2009; Tabernero and Woodman, 2018). This series of protein complexes includes the ESCRTs-0, -I and -II, ubiquitin-binding complexes, which sequester ubiquitylated cargo and then pass it to ESCRT-III (Frankel and Audhya, 2018; Henne et al., 2011; Piper et al., 2014). ESCRT-III is a generic membrane curvature-inducing polymer that combines with the downstream AAA ATPase VPS4 (VPS4A and VPS4B forms in mammals) to drive reverse topology membrane scission (Gatta and Carlton, 2019; Henne et al., 2013; McCullough et al., 2018; Pfitzner et al., 2021; Remec Pavlin and Hurley, 2020; Zhen et al., 2021), and thus at the MVB completes ILV formation (Raiborg and Stenmark, 2009; Vietri et al., 2020).

Early ESCRTs activate ESCRT-III assembly by recruiting the ESCRT-III nucleator subunit CHMP6, which subsequently promotes polymerisation of the core ESCRT-III component CHMP4B (Fyfe et al., 2011; Henne et al., 2012; Teis et al., 2010). CHMP4B polymerisation can also occur through a parallel pathway, involving Bro1 domain proteins. These conserved proteins (Odorizzi et al., 2003) contain a site within their Bro1 domains that binds to the C-terminus of CHMP4B (Kim et al., 2005), thereby relieving CHMP4B autoinhibition (Tang et al., 2016). The critical Bro1 protein directing MVB sorting of EGFR is His domain protein tyrosine phosphatase (HD-PTP; also known as PTPN23) (Doyotte et al., 2008), although its paralogue Alix (also known as PDCD6IP) is also important for EGFR degradation (Sun et al., 2015). The site in HD-PTP that engages CHMP4B also contributes to binding of the ESCRT-0 subunit STAM2 (Ali et al., 2013; Lee et al., 2016). Such competitive binding suggests that HD-PTP can facilitate movement of EGFR from early- to late-acting ESCRTs. Since HD-PTP also binds ESCRT-I (Stefani et al., 2011), HD-PTP scaffolds the MVB sorting machinery at multiple points.

MVB sorting begins within the early endosome, a compartment defined by the GTPase Rab5 (note herein, Rab4, Rab5 and Rab7 refer generically to all isoforms, with constructs and antibodies using Rab4a, Rab5a and Rab7a, respectively) (Wandinger-Ness and Zerial, 2014; Zerial and McBride, 2001; Zerial and Stenmark, 1993). The fully developed MVB fuses with the lysosome (Futter et al., 1996; Wartosch et al., 2015). However, this event occurs only after MVB sorting is complete, ensuring that bona fide lysosomal cargo, such as EGFR, is degraded efficiently, while also minimising the degradation of recycling cargo. The maturation of the MVB into a lysosomal fusion-competent late endosome involves replacing Rab5 with the late endosomal GTPase Rab7 (Huotari and Helenius, 2011; Rink et al., 2005). This process is termed ‘Rab conversion’ (Rink et al., 2005; Vonderheit and Helenius, 2005) and depends on the conserved Rab7 GEF complex Mon1–Ccz1, which activates Rab7 whilst also promoting Rab5 inactivation (Langemeyer et al., 2020; Nordmann et al., 2010; Poteryaev et al., 2010; Scott et al., 2014). A central question is whether Rab conversion is mechanistically coupled to the completion of MVB sorting. Studies in yeast suggest this may be the case, since ESCRT disruption traps MVB cargo in a ‘Class E’ compartment that contains hyperactivated Rab5 and fails to mature properly (Russell et al., 2012). However, the mechanisms linking MVB sorting to Rab conversion remain elusive.

The major Rab5 guanine nucleotide exchange factor (GEF) localised to the early endosome is Rabex-5 (also known as RABGEF1) (Horiuchi et al., 1997) and its essential partner Rabaptin-5 (also known as RABEP1) (Stenmark et al., 1995). The Rabex-5–Rabaptin-5 complex is decisive for controlling endosomal levels of GTP-Rab5 and promoting the formation of Rab5 domains (Cezanne et al., 2020; Langemeyer et al., 2014). Hence, coupling Rabex-5–Rabaptin-5 activity with the MVB sorting machinery could provide one mechanism that links the completion of MVB sorting to control of GTP-Rab5 levels and hence endosomal maturation.

Here, we demonstrate that HD-PTP links the ESCRT pathway to the Rabex-5–Rabaptin-5 complex by virtue of a direct interaction involving the CHMP4B and STAM2 binding interface in the HD-PTP Bro1 domain and Rabaptin-5. Consistent with its potential function as a molecular switch, loss of HD-PTP both prevents MVB sorting of EGFR and selectively elevates GTP-Rab5 levels to prevent endosomal maturation.

## RESULTS

### HD-PTP is required for EGFR activated by HB-EGF to exit Rab5 endosomes

**Fig. 1.**
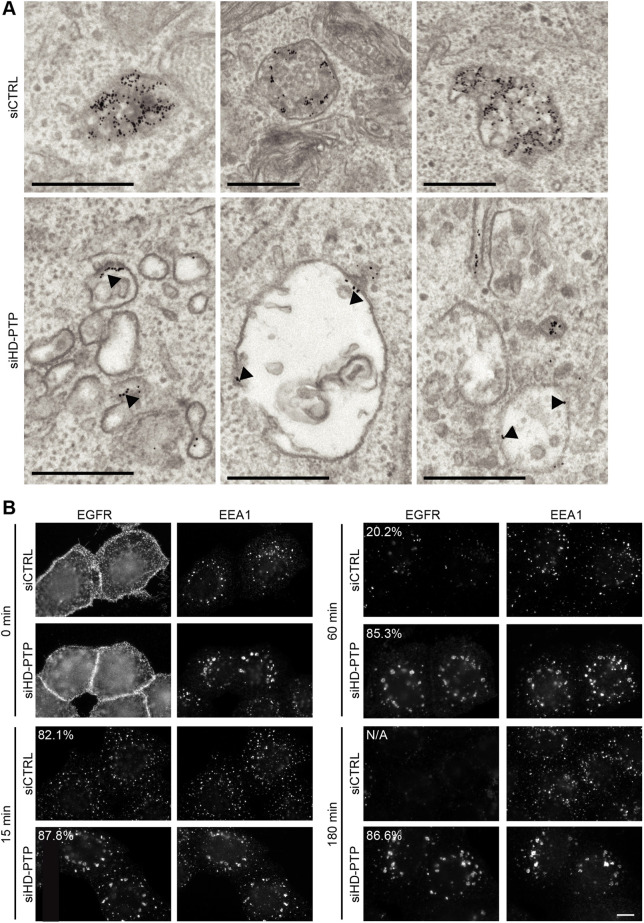
**HD-PTP regulates HB-EGF-induced EGFR sorting to the MVB and exit from EEA1-positive endosomes.** (A) Representative EM images of control (top) or HD-PTP-depleted (bottom) HeLa cells. Surface EGFR was labelled with gold-conjugated Mab108 and cells subsequently stimulated with HB-EGF for 30 min. Anti-EGFR-gold particles at the endosomal limiting membranes are indicated by arrowheads. Scale bars: 0.5 µm. Representative data from three independent experiments. (B) Control HeLa cells or cells depleted of HD-PTP were incubated with anti-EGFR antibody, stimulated with HB-EGF and chased for the indicated times, then immunostained. The percentages indicate the proportion of EGFR-containing structures that also labelled for EEA1. N/A, not applicable (i.e. very low EGFR signal). Representative data from three independent experiments. Scale bar: 10 µm

During normal EGF-induced receptor trafficking, a portion of EGFR enters the MVB pathway, while some EGFR is recycled (Bakker et al., 2017). We have previously reported that siRNA-mediated depletion of HD-PTP prevents lysosomal degradation of EGF (Doyotte et al., 2008) and inhibits the MVB sorting of EGFR stimulated by EGF (Ali et al., 2013). Here, we examined whether HD-PTP silencing also prevents the ILV sorting and exit from early endosomes of EGFR activated by heparin-binding EGF-like growth factor (HB-EGF), a ligand that exclusively sorts EGFR to the degradative pathway (Roepstorff et al., 2009). Cells were depleted of HD-PTP using low concentrations of siRNA for 48 h (siHD-PTP), conditions that led to the accumulation of ubiquitylated proteins on endosomes, a hallmark of loss of HD-PTP function (Doyotte et al., 2008) (Fig. S1A). Surface EGFR was labelled with gold-conjugated MAb108, an antibody that recognises the lumenal domain of EGFR but which does not impair ligand binding or receptor activation (Bellot et al., 1990; Felder et al., 1990). In control cells (siCTRL), EGFR stimulated with HB-EGF was sorted to the MVB lumen efficiently (Fig. 1A). In contrast, EGFR failed to sort to ILVs in HD-PTP-depleted cells, and instead accumulated within tubular-vesicular clusters and at the limiting membrane of neighbouring, often enlarged, endosomes (Fig. 1A). These data extend our previous findings that loss of HD-PTP impairs the MVB sorting of EGF-activated EGFR, as well as preventing the deubiquitylation of EGFR and lysosomal degradation of EGF and EGFR (Ali et al., 2013; Doyotte et al., 2008).

**Fig. 2.**
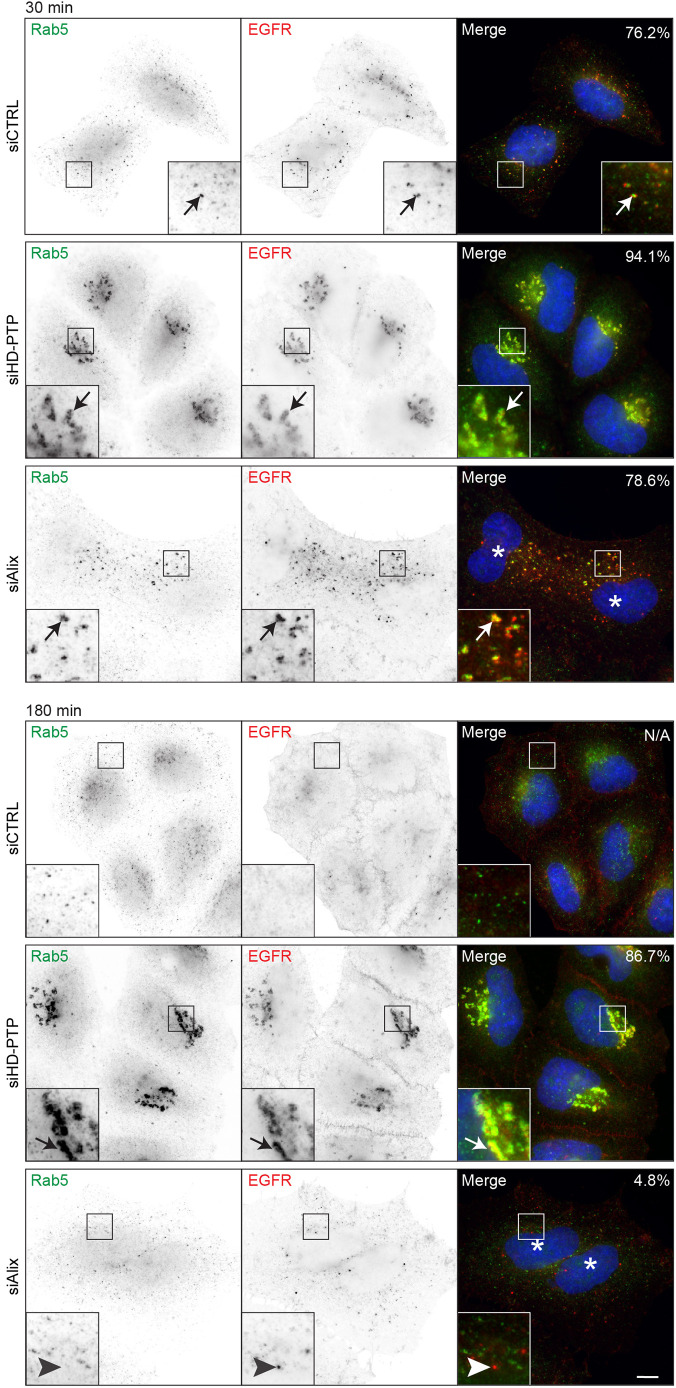
**EGFR activated by HB-EGF accumulation in Rab5-positive endosomes is specific to HD-PTP depletion.** Control HeLa cells or cells depleted of HD-PTP or Alix were incubated with fluorescently labelled EGFR, stimulated with HB-EGF, chased for up to 180 min and immunostained for Rab5. Arrows indicate colocalised Rab5 and EGFR puncta; arrowheads indicate undegraded EGFR not colocalised with Rab5-positive endosomes. Asterisks indicate nuclei from binucleate cells. Representative data from three independent experiments. The percentages indicate the proportion of EGFR-containing structures that also labelled for Rab5. Scale bar: 10 µm.

Fluorescence microscopy confirmed that HD-PTP is required to translocate HB-EGF-stimulated EGFR through early endosomes. In control cells, EGFR stimulated by HB-EGF was found in EEA1-positive endosomes within 15 min. EGFR staining diminished within 60 min and disappeared by 180 min, consistent with its lysosomal degradation. In HD-PTP-depleted cells, EGFR entered EEA1-positive endosomes, but failed to exit these clustered compartments (Fig. 1B). Rab5 staining confirmed that the compartments retaining EGFR were early endosomes (Fig. 2, arrows indicate EGFR located in Rab5-positive puncta), with Rab5 staining intensity significantly higher than in control cells. Failure of EGFR to transit Rab5-enriched early endosomes was not observed upon depletion of the HD-PTP paralogue Alix (Fig. S1B), despite Alix depletion reducing EGFR degradation, as reported previously (Doyotte et al., 2008; Sun et al., 2015). Transit of EGFR through Rab5-enriched endosomes was seen even in cells that were binucleate and thus clearly displaying an absence of Alix function (Carlton and Martin-Serrano, 2007; Morita et al., 2007) (Fig. 2, arrowheads indicate residual EGFR not colocalised in Rab5-positive endosomes). Thus, HD-PTP is essential both for MVB sorting and for the endosomal transit of HB-EGF-activated EGFR; these data are consistent with and expand upon our previous work (Ali et al., 2013).

### Depletion of HD-PTP enhances Rab5 activity on endosomes

The failure of cargo to exit early endosomes suggested that HD-PTP depletion might cause a deficit in Rab5 inactivation, an event that is essential for maturation of the early endosome to a late endosome (Rink et al., 2005). The Rab5 GEF Rabex-5, and the Rab5 effector Rabaptin-5, form a complex that determines most of the Rab5 activity at early endosomes (Cezanne et al., 2020; Horiuchi et al., 1997; Lauer et al., 2019; Zhang et al., 2014). Thus, we examined whether HD-PTP controls Rabex-5–Rabaptin-5 function. For these experiments, we examined unstimulated cells, to eliminate any compounding effects of EGFR activation on Rab5 activity.

**Fig. 3.**
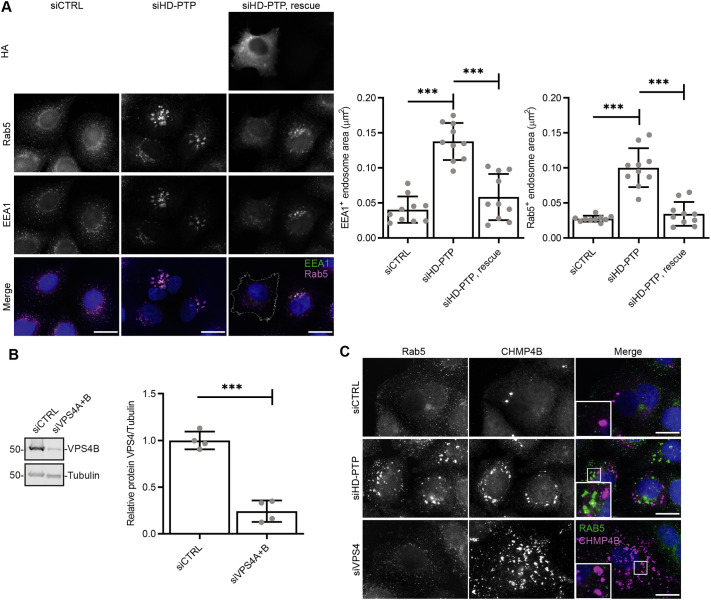
**The effect of HD-PTP depletion on endosomal clustering is not secondary to ESCRT depletion.** (A) Endosomal clusters induced by HD-PTP depletion is not an off-target effect. HeLa cells were depleted with siRNA and rescued with RNAi-resistant HA–HD-PTP as indicated and immunostained for Rab5 and EEA1. Graph shows quantification of the approximate area of endosomal structures. Values represent mean±s.d. (*n*=10). ***P<0.0001 (one-way ANOVA with Bonferroni's test for multiple comparisons). (B) Validation of siRNA silencing of VPS4B. Values represent mean±s.d. (*n*=4). ***P<0.0001 (unpaired two-tailed Student's *t*-test). (C) Aberrant ESCRT-III accumulation caused by HD-PTP depletion is not responsible for clustering of Rab5 endosomes. HeLa cells were depleted with siRNA as indicated and immunostained for Rab5 and CHMP4B. Representative data from three independent experiments. Scale bars: 20 µm.

**Fig. 4.**
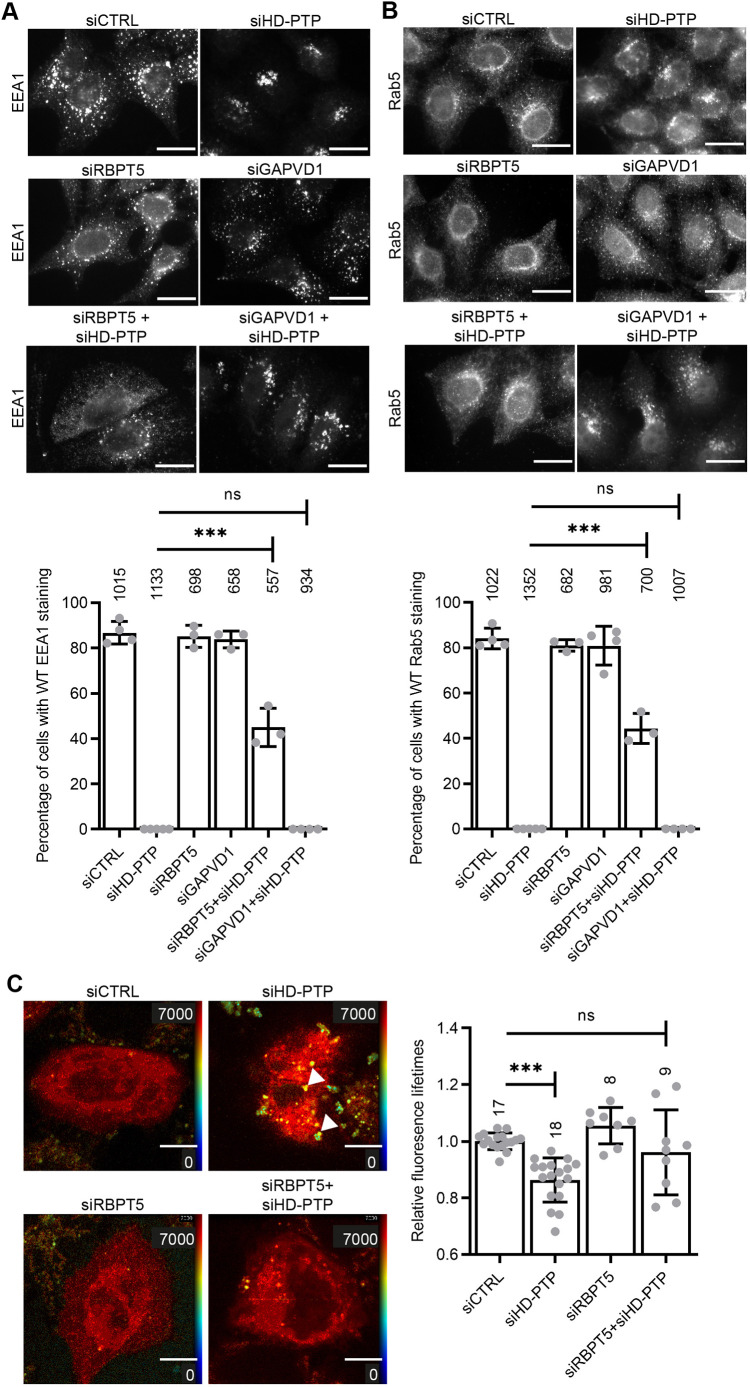
**Depletion of HD-PTP enhances Rab5 activity on endosomes.** (A) HeLa cells were depleted with siRNA as indicated and immunostained for EEA1. Histogram shows the percentage of cells that displayed a normal distribution of EEA1 (mean±s.d. from at least three independent experiments, 100–300 cells counted per experiment, total number of cells indicated above each histogram bar). ***P<0.0001; ns, not significant (one-way ANOVA with Bonferroni's test for multiple comparisons). RBPT5, Rabaptin-5. (B) As A, but immunostained for Rab5. (C) HeLa cells were depleted with siRNA as indicated and transfected with NowGFP–Rab5a and mRuby–EEA1.sh-R5BD. The fluorescence lifetime of NowGFP–Rab5a was measured. Panels show the scale and distribution of fluorescence lifetime in representative cells (lookup chart key on right, black/blue=0 ns, red=7000 ns). Arrowheads indicate pockets of decreased fluorescence lifetime. Graph shows quantification of fluorescence lifetimes over whole cell areas. Values represent mean±s.d. (*n*=8–18 cells from three independent experiments, total number of cells indicated above each histogram bar). ***P<0.0001, ns, not significant (one-way ANOVA with Bonferroni's test for multiple comparisons). Scale bars: 20 µm.

As expected, HD-PTP depletion generated clustered EEA1-positive endosomes that labelled strongly for Rab5, a phenotype that was fully rescued by expression of RNAi-resistant HA-HD-PTP (Fig. 3A). The concentration of Rab5 on endosomes was not merely due to a defective ESCRT pathway, since depletion of VPS4A and VPS4B (siVPS4A+B) (Fig. 3B) did not give rise to such intense Rab5 staining despite, like HD-PTP depletion, inducing clustering of the ESCRT-III subunit CHMP4B (Fig. 3C). Notably, Rab5 did not colocalise with CHMP4B clusters in HD-PTP-depleted cells (Fig. 3C), consistent with the inability of endocytic cargo to reach CHMP4B in the absence of HD-PTP (Ali et al., 2013) and further suggesting that Rab5 accumulation in HD-PTP-depleted cells is not merely an indirect product of blocked ESCRT-III function. Co-depletion of Rabaptin-5 (Fig. S1C; unfortunately, we could not obtain consistent knockdown of Rabex-5) largely prevented the clustering of EEA1 and Rab5 induced by HD-PTP depletion (Fig. 4A,B). In contrast, co-depletion of GAPVD1 (Fig. S1D), a Rab5 GEF acting at an earlier point of the endocytic pathway (Sato et al., 2005; Semerdjieva et al., 2008), had no effect (Fig. 4A,B).

To test directly whether levels of active (i.e. GTP-bound) Rab5 on endosomes were enhanced by loss of HD-PTP, we conducted FRET-FLIM experiments using Now-GFP–Rab5a and a mRUBY-tagged Rab5-binding domain from EEA1 (EEA1.sh-R5BD), based on previously characterised reporters (Galperin and Sorkin, 2005). Interaction between these probes results in transfer of energy from excited GFP to non-excited mRUBY, leading to increased mRUBY fluorescence and a decrease in the lifetime of GFP fluorescence. GFP fluorescence lifetimes were decreased (Fig. 4C, histogram) in cells depleted of HD-PTP, demonstrating an increased interaction of Now-GFP–Rab5a with mRUBY–EEA1.sh-R5BD, with the FRET signal highest over cytoplasmic puncta (Fig. 4C panel, arrowheads). Hence, HD-PTP depletion enhances endosomal Rab5 activity. These effects were largely reversed upon co-depletion of Rabaptin-5 (Fig. 4C). In summary, the increased level of endosomal GTP-Rab5 in HD-PTP-depleted cells relies on Rabex-5–Rabaptin-5.

### Rabaptin-5 is hyperphosphorylated in HD-PTP-depleted cells

**Fig. 5.**
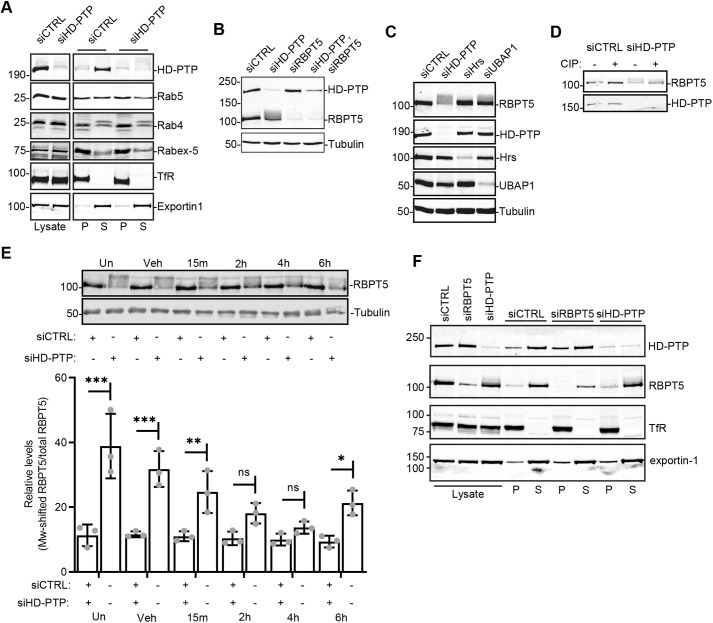
**Rabaptin-5 is hyperphosphorylated in HD-PTP-depleted cells.** (A) Lysates from HeLa cells depleted of HD-PTP or control cell lysates (left) were fractionated (right; P, pellet; S, supernatant) and proteins detected by western blotting. Representative data from three independent experiments. (B,C) Lysates from HeLa cells depleted with siRNA as indicated were analysed by western blotting. Representative data from three independent experiments. RBPT5, Rabaptin-5. (D) Lysates prepared from control or HD-PTP-depleted HeLa cells were treated with CIP (+) or vehicle control (−) and endogenous Rabaptin-5 analysed by western blotting. Representative data from three independent experiments. (E) HeLa cells were siRNA depleted as indicated and treated with CID755673 for the indicated time or vehicle control (Veh), or left untreated (Un). Lysates were harvested and proteins detected by western blotting. Graph shows quantification of total molecular mass-shifted Rabaptin-5 levels. Values represent mean±s.d. (*n*=3). ***P<0.0001; **P<0.01; *P<0.05; ns, not significant (two-way ANOVA with Sidak's test for multiple comparisons). (F) Lysates from HeLa cells depleted of HD-PTP or Rabaptin-5, or control cell lysates, were fractionated (P, pellet; S, supernatant) and proteins detected by western blotting. Representative data from three independent experiments.

A simple explanation for higher levels of active Rab5 in HD-PTP-depleted cells could be increased levels of Rab5 protein. However, levels of Rab5, or of Rab4, which works upstream of Rabaptin-5 (Kalin et al., 2015), did not alter upon HD-PTP depletion (Fig. 5A,B). A further explanation could be the increased pool of ubiquitylated proteins associated with endosomes upon HD-PTP depletion (Doyotte et al., 2008), since ubiquitin binding helps recruit Rabex-5 to endosomes (Mattera and Bonifacino, 2008) and also increases its Rab5 GEF activity modestly (Lauer et al., 2019). However, the majority of Rabex-5 was membrane associated even in control cells, and no further increase was detected upon HD-PTP depletion (Fig. 5A). We envisaged instead that HD-PTP might control Rabex-5–Rabaptin-5 directly. Consistent with such an effect, the migration of endogenous Rabaptin-5 on SDS-PAGE gels shifted quantitatively to higher molecular mass form(s) in HD-PTP-depleted cells (Fig. 5B). This shift was particularly evident when SDS-PAGE samples were prepared without boiling, which is more likely to preserve post-translational modification(s). The effect was not observed upon depletion of the ESCRT-0 subunit Hrs (also known as HGS) (Fig. 5C). Depletion of the ESCRT-I subunit UBAP1, which interacts with HD-PTP (Stefani et al., 2011), produced a modest change in Rabaptin-5 migration (Fig. 5C).

This molecular mass shift is due at least in part to Rabaptin-5 phosphorylation, since it was reduced when cell extracts were treated with calf intestinal phosphatase (CIP) prior to SDS-PAGE (Fig. 5D). Rabaptin-5 has previously been reported as a substrate for protein kinase D (PKD) in migrating cells (Christoforides et al., 2012). We tested whether PKD might contribute to the enhanced phosphorylation of Rabaptin-5 that we observed, by treating cells with the selective PKD inhibitor CID755673 (Sharlow et al., 2008). Indeed, incubating HD-PTP-depleted cells with CID755673 for >2 h reduced the MW shift in Rabaptin-5 significantly (Fig. 5E). The effect of HD-PTP depletion on Rabaptin-5 phosphorylation did not correlate with altered membrane association, since the distribution of Rabaptin-5 was unaltered upon loss of HD-PTP (Fig. 5F). Altogether, these data suggest that HD-PTP may influence the catalytic cycle of Rabex-5–Rabaptin-5, rather than its assembly onto endosomes.

### HD-PTP binds to Rabaptin-5 CC2-2 to control endosomal properties

**Fig. 6.**
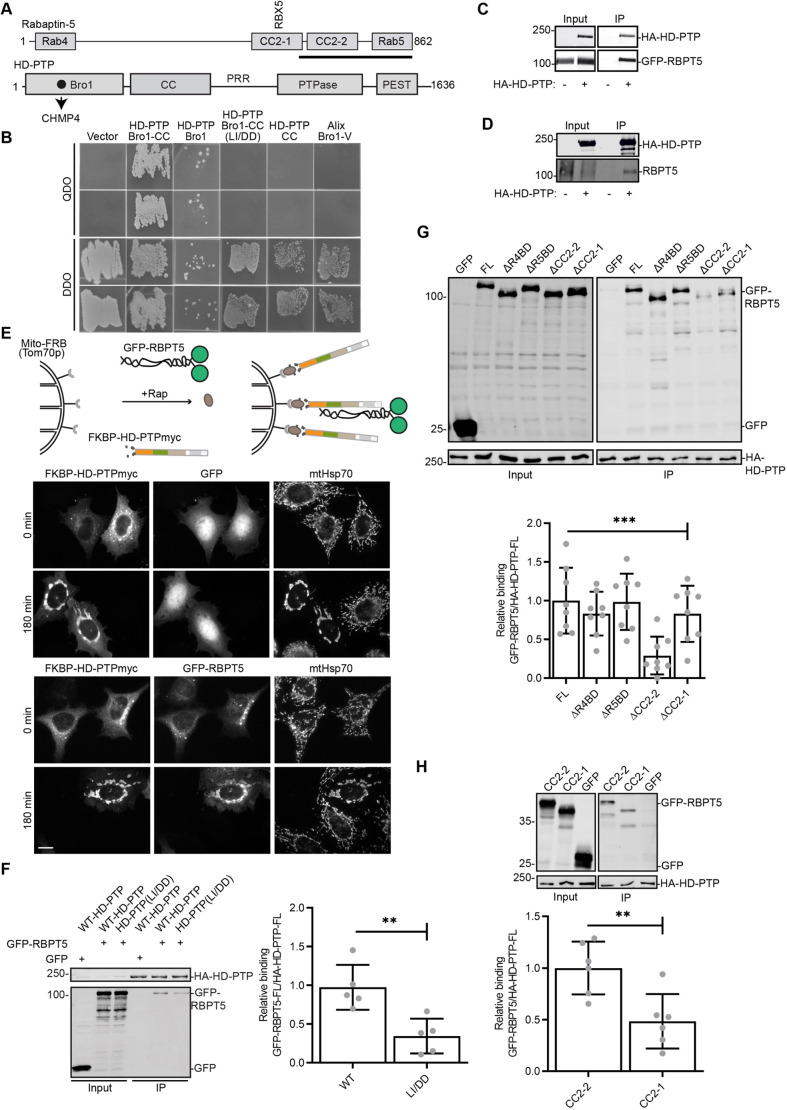
**HD-PTP and Rabaptin-5 interact via the ESCRT-III-binding site in HD-PTP and Rabaptin-5 CC2-2.** (A) Schematic representation of key Rabaptin-5 and HD-PTP domains and interacting regions. The CHMP4/ESCRT-III-binding site in HD-PTP is highlighted by a dark circle. The minimal interacting region of Rabaptin-5 (RBPT5) identified by Y2H screen is underlined in bold. (B) Yeast cultures were co-transformed with full-length Rabaptin-5 and with the indicated HD-PTP constructs, and liquid cultures were streaked onto agar plates containing selection media as shown. DDO refers to double dropout medium, QDO refers to quadruple dropout medium. Representative data from three independent experiments. (C) Lysates from HEK293T cells co-transfected with GFP-Rabaptin-5 and HA-HD-PTP were immunoprecipitated (IP) with anti-HA and proteins detected by western blotting. Input is 2% of offered protein. Representative data from three independent experiments. (D) Lysates from HEK293T cells transfected with HA–HD-PTP were immunoprecipitated with anti-HA and proteins detected by western blotting. Input is 2% of offered protein. Representative data from three independent experiments. (E) Relocated HD-PTP recruits GFP–Rabaptin-5 to mitochondria. Top, schematic showing FKBP–HD-PTP–Myc relocating to mitochondria upon rapamycin-induced heterodimerisation of FKBP with the mitochondrially localised Mito-FRB. Bottom, immunofluorescence of HeLa cells expressing Mito-FRB, FKBP–HD-PTP–Myc and either GFP or GFP–Rabaptin-5 treated with rapamycin for 180 min or vehicle control (0 min). Cells were co-stained for the Myc epitope and mitochondrial marker mtHsp70. Scale bar: 10 µm. Representative data from three independent experiments. (F) Lysates from HEK293T cells co-transfected with GFP-Rabaptin-5 and WT or L202D/I206D (LI/DD) HA–HD-PTP were immunoprecipitated with anti-HA and proteins detected by western blotting. Input is 2% of lysate. Graph shows quantification of GFP–Rabaptin-5 binding to HA-HD-PTP, values represent mean±s.d. (*n*=5). **P<0.01 (one-way ANOVA with Bonferroni's test for multiple comparisons). (G) Lysates from HEK293T cells transfected with GFP–Rabaptin-5 truncation mutants and HA–HD-PTP were immunoprecipitated with anti-HA and proteins detected by western blotting. Input is 2% of lysate. Graph shows quantification of GFP-Rabaptin-5 binding to HA–HD-PTP. Values represent mean±s.d. (*n*=8). ***P<0.001 (one-way ANOVA with Bonferroni's test for multiple comparisons). (H) Lysates from HEK293T cells co-transfected with GFP–Rabaptin-5 fragments and HA–HD-PTP were immunoprecipitated with anti-HA and proteins detected by western blotting. Input is 2% of lysate. Graph shows quantification of GFP–Rabaptin-5 binding to HA–HD-PTP. Values represent mean±s.d. (*n*=6). **P<0.01 (unpaired two-tailed Student's *t*-test).

Consistent with the effect of HD-PTP depletion on Rabaptin-5, a yeast two-hybrid (Y2H) screen employing as bait the minimal essential fragment of HD-PTP, Bro1CC (Ali et al., 2013; Gahloth et al., 2017), identified 7 Rabaptin-5 clones, with a minimal binding region of Rabaptin-5 residues 630–862 (Fig. 6A). Directed Y2H confirmed the interaction, and also showed that it was selective for HD-PTP over Alix (Fig. 6B). Importantly, binding involved the HD-PTP Bro1 domain and was abolished by a L202D/I206D double mutation (HD-PTP^LI/DD^) (Fig. 6B), which disrupts the CHMP4B-binding pocket in HD-PTP, a key site for driving the MVB sorting function of HD-PTP (Ali et al., 2013; Doyotte et al., 2008). Consistent with an interaction at this site, wild-type (WT) HA–HD-PTP but not HA–HD-PTP^L202D^ colocalised strongly with GFP–Rabaptin-5 and endogenous Rab5 (Fig. S2A), in contrast to the modest recruitment of HA–HD-PTP to Rab5- positive endosomes in the absence of exogenous GFP–Rabaptin-5 (Fig. S2B). Furthermore, HD-PTP^LI/DD^ could not rescue the enlargement and clustering of endosomes caused by HD-PTP depletion (Fig. S2C), highlighting the importance of this site for controlling endosome maturation and in line with it binding to Rabaptin-5.

Association of full-length HA–HD-PTP with endogenous Rabaptin-5, as well as with GFP–Rabaptin-5, was confirmed by their co-immunoprecipitation from cell extracts (Fig. 6C,D). Binding of Rabaptin-5 to HD-PTP was also confirmed using a mitochondrial-targeting assay (Silvius et al., 2006), in which cells co-expressed an FKBP12-Rapamycin binding (FRB) construct targeted to the mitochondrial outer membrane (mito-FRB) and FKBP12–HD-PTP–Myc. Rapamycin caused FKBP12–HD-PTP to relocate to mitochondria that became highly clustered (Fig. 6E), while also relocalizing GFP–Rabaptin-5 but not GFP (Fig. 6E). As anticipated, the association between HD-PTP and Rabaptin-5 was reduced significantly, though not abolished, by mutation of the CHMP4/ESCRT-III binding pocket in HD-PTP (Fig. 6F).

We mapped the association within Rabaptin-5 by co-expressing targeted deletions of GFP–Rabaptin-5 (Fig. 6G). Since Rabaptin-5 is a homodimer (Stenmark et al., 1995), we used HEK293T cells because these expressed GFP–Rabaptin-5 at high levels. These high levels of GFP–Rabaptin-5, compared to endogenous protein, minimised any potential for dimerisation between GFP–Rabaptin-5 and endogenous Rabaptin-5 that might complicate analysis. Rabaptin-5 (Fig. 6A) contains a Rab4-binding domain [amino acids (aa) 5–135] and a C-terminal Rab5-binding domain (aa 815–862) (Kalin et al., 2015; Zhu et al., 2004) and contains several known or predicted coiled coil-forming helices. Notably, CC2-1 (aa 552–642) binds Rabex-5 and helps to expose the Rabex-5 catalytic site to Rab5 (Zhang et al., 2014). The minimal binding region found in the Y2H screen encompasses a predicted coiled coil-forming region, CC2-2 (aa 661–805), lying between the C-terminal Rab5-binding site and the Rabex-5 interaction domain (Fig. 6A). Co-immunoprecipitation experiments showed that deletion of the indicated Rab4- or Rab5-binding domains did not affect the interaction of GFP–Rabaptin-5 with HA–HD-PTP (Fig. 6G). In contrast, deletion of CC2-2 markedly reduced binding of GFP–Rabaptin-5 to HD-PTP (Fig. 6G). Binding of GFP–Rabaptin-5 to HD-PTP was also reduced slightly, though not significantly, by deletion of CC2-1 (Fig. 6G). Further experiments confirmed that the Rabaptin-5 CC2-2 domain alone interacts with HD-PTP (Fig. 6H). Rabaptin-5 CC2-1 also bound to HD-PTP, albeit less well (Fig. 6H).

### Rabaptin-5 binds directly to the HD-PTP ‘S’ pocket, competing with ESCRT-III

**Fig. 7.**
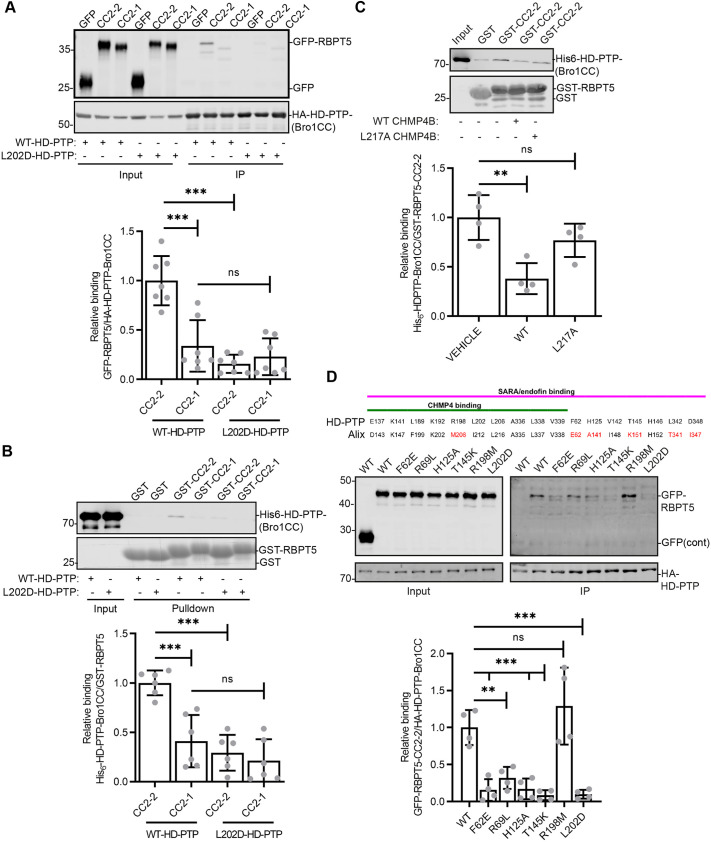
**Rabaptin-5 CC2-2 binds directly to the CHMP4-binding and neighbouring ‘S’ pockets of HD-PTP.** (A) Lysates from HEK293T cells transfected with GFP–Rabaptin-5 (RBPT5) truncation mutants and WT or L202D HA–HD-PTP-Bro1CC were immunoprecipitated (IP) and proteins detected by western blotting. Input is 2% of offered protein. Graph shows quantification of GFP-Rabaptin-5 binding to HA–HD-PTP. Values represent mean±s.d. (*n*=7). ***P<0.0001; ns, not significant (one-way ANOVA with Bonferroni's test for multiple comparisons). (B) Recombinant GST–Rabaptin-5 truncation mutants and WT or L202D His_6_–HD-PTP-Bro1CC were incubated with glutathione agarose beads and proteins detected by western blotting. Input is 3% of offered protein. Graph shows quantification of GST–Rabaptin-5 binding to His_6_–HD-PTP-Bro1CC. Values represent mean±s.d. (*n*=6). ***P<0.001 (one-way ANOVA with Bonferroni's test for multiple comparisons). (C) Recombinant GST–Rabaptin-5-CC2-2 and WT His_6_–HD-PTP-Bro1CC were incubated with WT or L217A CHMP4B peptide (aa 205–224), immobilised on glutathione agarose beads and proteins detected by western blotting. Input is 3% of offered protein. Graph shows quantification of GST–Rabaptin-5 binding to His_6_–HD-PTP-Bro1CC. Values represent mean±s.d. (*n*=4). **P<0.01; ns, not significant (one-way ANOVA with Bonferroni's test for multiple comparisons). (D) Residues in Bro1 domains involved in binding to effector peptides. Conserved residues are in black, residues that differ in Alix are in red. Lysates from HEK293T cells transfected with GFP–Rabaptin-5-CC2-2 and WT, F62E, R69L, H125A, T145K, R198M or L202D HA–HD-PTP-Bro1CC were immunoprecipitated and proteins detected by western blotting. Input is 2% of offered protein. Graph shows quantification of GFP–Rabaptin-5 binding to HA–HD-PTP. Values represent mean±s.d. (*n*=4). ***P<0.001; **P<0.01; ns, not significant (one-way ANOVA with Bonferroni's test for multiple comparisons).

Both CC2-2, and to a small extent CC2-1, interacted with the isolated Bro1CC fragment of HD-PTP (Fig. 7A). Binding is direct, since bacterially expressed CC2-2 (and to a more limited extent CC2-1) bound to bacterially expressed HD-PTP-Bro1CC (Fig. 7B). In cells, as well as between bacterially expressed proteins, binding of the Rabaptin-5 CC2-2 domain to HD-PTP was substantially reduced by mutation of the CHMP4B-binding pocket (Fig. 7A,B). In contrast, the modest binding of Rabaptin-5 CC2-1 to HD-PTP–Bro1CC was not affected significantly by this mutation. The interaction between Rabaptin-5 CC2-2 and HD-PTP Bro1CC recombinant proteins could be competed off with a WT CHMP4B peptide, but not a mutant CHMP4B peptide (McCullough et al., 2008) that cannot bind HD-PTP (Gahloth et al., 2017; McCullough et al., 2008) (Fig. 7C).

The CHMP4-binding region of HD-PTP is distinguished from that of Alix by the presence adjacent to the CHMP4 binding pocket of an ‘S’, or ‘specificity’, pocket (Gahloth et al., 2017). The S pocket, together with the CHMP4-binding pocket, forms a contiguous interface that accommodates the endosomal SMAD regulators endofin and SARA (also known as ZFYVE16 and ZFYVE9, respectively) (Gahloth et al., 2017), and which is partially occupied by the ESCRT-0 subunit STAM2 (Lee et al., 2016). These endosomal proteins compete with CHMP4 for binding to HD-PTP (Ali et al., 2013; Gahloth et al., 2017), and as such permit the coordination of ESCRT-III assembly with other early endosome functions. Since HD-PTP but not Alix controls endosomal maturation, we tested whether Rabaptin-5 binding also involves the S pocket. Several residues in the HD-PTP S pocket are important for endofin interaction (Gahloth et al., 2017) (Fig. 7D). One critical residue is T145. The corresponding residue in Alix is K151, the bulky side chain of which prevents peptide ligands entering the S pocket region. Within the S pocket, endofin contacts F62 and H125, while also being close to R69. A further residue, R198, lies in the CHMP4 pocket but is reoriented upon endofin binding. Mutation of F62, H125 and T145 strongly affects HD-PTP binding to endofin, whereas mutation of R69 and R198 has a more modest effect (Gahloth et al., 2017). Here, we find that mutation of F62, R69, H125 and T145 all strongly impaired the binding of HD-PTP to Rabaptin-5 (Fig. 7D), indicating that the S pocket is utilised, although the contacts with Rabaptin-5 may differ slightly from those with endofin.

In summary, HD-PTP controls ESCRT assembly and Rab5 activity. Although there might be ancillary interaction(s) involving other site(s), the major interaction between HD-PTP and Rabaptin-5 involves the CHMP4/ESCRT-III binding and neighbouring ‘S’ pockets within HD-PTP, and the Rabaptin-5 CC2-2 domain. HD-PTP is thus positioned to coordinate MVB sorting with Rab5 activity.

## DISCUSSION

We have identified and characterised a direct interaction between HD-PTP and Rabaptin-5 and have dissected its role in endosomal function. Specifically, we find that HD-PTP acts through Rabex-5–Rabaptin-5 to influence endosomal maturation and thus coordinate this process with MVB sorting.

We previously showed that HD-PTP is essential for the correct MVB sorting of EGF-activated EGFR (Ali et al., 2013; Doyotte et al., 2008). Here, we extend these findings for EGFR stimulated by HB-EGF, which more strongly targets EGFR for MVB sorting and degradation, while also showing that the early endosomes in which EGFR becomes trapped contain concentrated and hyperactivated Rab5. This effect on endosomal trafficking was not observed upon depletion of the paralogous protein Alix. Alix binds directly to and regulates the sorting of other cargo, such as GPCRs (Dores et al., 2012) and exosome cargo, including from the late endosome (Larios et al., 2020). Hence, distinct endosomal cargoes recruit different interacting partners to determine their trafficking fate, with HD-PTP linking MVB sorting to exit from early endosomes.

The persistence of degradative cargo within Rab5-enriched compartments in HD-PTP-depleted cells indicates that HD-PTP may facilitate efficient endosomal maturation to late endosomes, thereby negatively regulating early endosome identity. One of the best-studied mechanisms of maintaining early endosomes is the Rabaptin-5-dependent feedback loop that ensures full enhancement of Rab5 activity. The Rabex-5–Rabaptin-5 complex is recruited to early endosomes via Rab4 and ubiquitylated cargo (Kalin et al., 2015; Lauer et al., 2019; Mattera and Bonifacino, 2008; Penengo et al., 2006), thereafter driving cycles of Rab5 activation (Zhang et al., 2014) and further Rabex-5–Rabaptin-5 recruitment to generate membrane domains with a high local content of GTP-Rab5 (Cezanne et al., 2020) and thus an enrichment of Rab5 effector proteins (McBride et al., 1999; Zerial and McBride, 2001). It is this positive feedback cycle that HD-PTP appears to disrupt, by directly binding Rabaptin-5. Notably, this interaction involves the HD-PTP ‘S’ (specificity) pocket, which is adjacent to the generic CHMP4-binding site found in other Bro1 proteins but which is found only in HD-PTP (Gahloth et al., 2017). The S pocket also accommodates the endosomal proteins endofin and SARA (Gahloth et al., 2017), and is partially occupied by the ESCRT-0 component STAM2 (Lee et al., 2016), highlighting HD-PTP as an early endosome-specific ESCRT regulator.

HD-PTP may control Rabaptin-5 function through several mechanisms. Our data suggest that HD-PTP opposes PKD-mediated phosphorylation of Rabaptin-5. Previous studies have reported that PKD-mediated phosphorylation of Rabaptin-5 promoted Rab4-dependent platelet-derived growth factor receptor (PDGFR) recycling (Christoforides et al., 2012), and it is notable that HD-PTP drives PDGFR as well as EGFR degradation (Ma et al., 2015). Our study is consistent with the underlying premise that PKD-dependent phosphorylation of Rabaptin-5 opposes receptor degradation, whereas HD-PTP promotes Rabaptin-5 dephosphorylation. It is possible that HD-PTP phosphatase activity directly opposes PKD activity, but this is unlikely given that HD-PTP is a tyrosine phosphatase family member, and indeed may lack phosphatase activity entirely (Barr et al., 2009; Gingras et al., 2009). More likely, HD-PTP regulates signalling pathways that control PKD activity locally, or sterically influences PKD-dependent Rabaptin-5 phosphorylation.

HD-PTP may also inhibit Rabaptin-5 function directly, by binding to the CC2-2 region. CC2-2 does not interact with Rabex-5, so is unlikely to be involved in promoting guanine nucleotide exchange on Rab5. However, CC2-2 lies between the Rabex-5-binding region (CC2-1) and the C-terminal Rab5-binding site, and as such may be critical for relaying the ‘handover’ of Rab5 that underpins the positive feedback loop allowing Rabex-5–Rabaptin-5 to drive the development of Rab5-positive endosomal domains (Cezanne et al., 2020). Future work will assess precisely how the HD-PTP S pocket (Gahloth et al., 2017) engages Rabaptin-5, and the impact of this interaction on Rabaptin-5 function. The binding of HD-PTP to CC2-1, albeit more modest than the binding to CC2-2, may also influence Rabex-5-dependent guanine nucleotide exchange on Rab5.

**Fig. 8.**
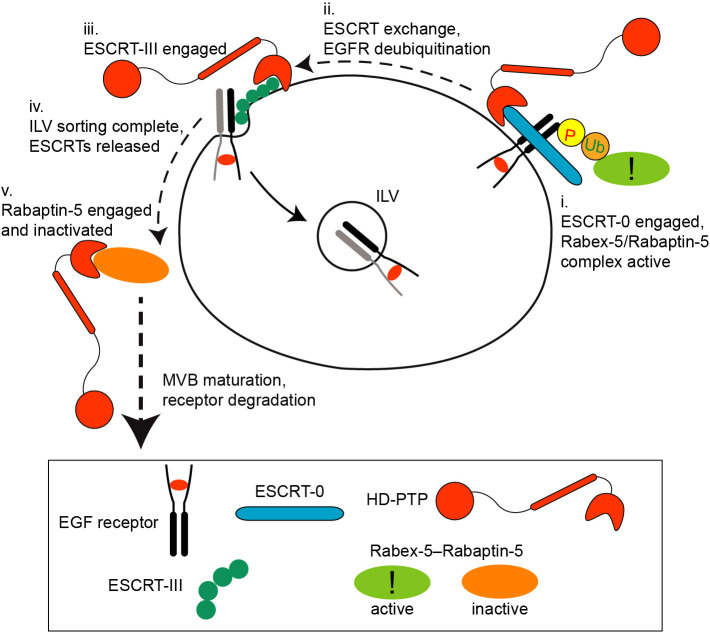
**Model of the coupling of endosomal sorting and maturation.** (i) HD-PTP initiates ESCRT-dependent MVB sorting of cargo by binding STAM2/ESCRT-0 while Rabex-5–Rabaptin-5 engages ubiquitylated cargo and is active to maintain high GTP-Rab5. (ii,iii) As cargo sorting proceeds, HD-PTP releases STAM2/ESCRT-0 and then binds CHMP4/ESCRT-III, while cargo is deubiquitylated. (iv) Cargo enters ILVs as CHMP4B/ESCRT-III is released. (v) HD-PTP, now free from ESCRTs, bind to Rabaptin-5. Rabex-5–Rabaptin-5 is inhibited, thus lowering GTP-Rab5 and promoting Rab conversion.

HD-PTP engages ubiquitylated cargo at the early endosome in coordination with ESCRT-0, as a prelude to ESCRT-III binding (Ali et al., 2013). Hence, it seems likely that while MVB sorting is incomplete, HD-PTP is engaged with ESCRT-I and ESCRT-III, and thus not available to bind Rabaptin-5. This would allow Rabaptin-5 to maintain Rab5 activation while ILV sorting continues (Fig. 8). Indeed, studies using *S. cerevisiae* show that active Rab5 is important for ILV sorting to continue, and that Rab5 activity is maintained by the Rabex-5 orthologue Vps9 (Shideler et al., 2015). Presumably, high levels of GTP-Rab5 help, via VPS34, to maintain levels of phosphatidylinositol-3-phosphate (PI3P), which is essential for ESCRT-0 recruitment (Futter et al., 2001). Once cargo sorting is complete (and ESCRTs are consequently released from the endosome membrane), HD-PTP would become available to bind and inhibit Rabaptin-5 (Fig. 8). Such a mechanism would ensure that early endosomes can only begin their maturation into late endosomes when ILV sorting is complete. It is likely that the cargo deubiquitylation that accompanies cargo sorting also contributes to reduced Rabex-5–Rabaptin-5 function (Lauer et al., 2019; Mattera and Bonifacino, 2008; Penengo et al., 2006; Russell et al., 2012; Shideler et al., 2015) and thereby further decreases Rab5 activity. Hence, HD-PTP could dually regulate Rab5 levels by direct and indirect pathways that converge on Rabex-5–Rabaptin-5.

Endosomal maturation dictates that Rab5 must be lost from early endosomes in favour of Rab7 in a process known as Rab conversion (Rink et al., 2005). The Mon1–Ccz1 complex helps achieve this by recruiting the HOPS Rab7 GEF complex, while also promoting the release from endosomes of Rabex-5 (Poteryaev et al., 2010; Scott et al., 2014). We propose that HD-PTP works alongside Mon1–Ccz1 via encouraging a reduction of Rabaptin-5 function and subsequent decrease in Rab5 activity. It remains to be elucidated how these two pathways for Rab conversion function together to control maturation. However, HD-PTP is likely to serve as an important molecular check to ensure that cargo is properly sorted into ILVs, whereas Mon1–Ccz1 responds to threshold levels of Rab5 and/or PI3P to sense that maturation can occur (Poteryaev et al., 2010). Such complementary mechanisms would optimise the timing of endosomal maturation versus completion of early endosome function.

Rab activity is upregulated in a variety of cancers (Tzeng and Wang, 2016); indeed Rab5 is no exception and its activity is enhanced in lung and breast cancers (Frittoli et al., 2014; Silva et al., 2016). In general, increased cell motility is thought to occur via persistent receptor signalling, caused by attenuated degradation. Moreover, both Rabaptin-5 and HD-PTP are known tumour suppressors (Gingras et al., 2017; Thomas and Strutt, 2014), and changes in their protein expression lead to prolonged receptor activity. Therefore, understanding the HD-PTP and Rabaptin-5 interaction might provide crucial insight into how endosomal function influences cancer progression.

## MATERIALS AND METHODS

### Reagents

#### Antibodies

Anti-EGFR antibody, MAb 108, was purified from supernatants of the hybridoma cell line HB-9764 (ATCC) to 1 mg/ml and used at 1:2000 for immunofluorescence (IF) (Stefani et al., 2011). The following commercial antibodies were used. Mouse: anti-Hrs clone A-5 [Enzo Life Sciences; Cat. ALX-804-382-C050; batch 02021715; 1:2500 for western blotting (WB)]; FK2 anti-ubiquitin (Enzo Life Sciences; Cat. BML-PW-8810-0500; batch 07241306; 1:50 for IF); anti-HA (GenScript; Cat. A01244; batch A204810; 1:2500 for WB, 1:1000 for IF); anti-EEA1 (BD Biosciences; 14/EEA1; Cat. 610457; batch 6302827; 1:400 for IF); anti-Rab5 (BD Biosciences; Cat. 610725; batch 11971; 1:500 for IF; 1:2500 for WB); anti-TfR (Zymed; Doyotte et al., 2008); anti-His_6_ clone H1029 (Sigma; Cat. 062M4809; 1:5000 for WB); anti-mtHsp70 clone JG-1 (Thermo Scientific - Pierce; Cat. Ma3 028; batch PK209570 1:250 for IF); anti-Myc clone 9E10 (Millipore; Cat. M5546; batch 075K4755; 1:50 for IF). Rabbit: anti-CHMP4B (Proteintech; Cat. 13683-1-AP; batch 00019545; 1:250 for IF); anti-HD-PTP (Proteintech; Cat. 10472-1-AP; batch 00018154; 1:5000 for WB; 1:50 for IF) (Stefani et al., 2011); anti-UBAP1 (Proteintech; Cat. 12385-1-AP; batch 00014891) (Stefani et al., 2011); anti-EEA1 (Cell Signaling Technologies; C45B10; Cat. 3288S; batch 8; 1:400 for IF); anti-Rabex-5 (Thermo Scientific - Invitrogen; Cat. PA5-21117; batch RF2221849; 1:2500 for WB); anti-Rabaptin-5 (Proteintech; Cat. 14350-1-AP; batch 00005401; 1:5000 for WB); anti-Rab5 clone C8B1 (Cell Signaling Technologies; Cat. 3547S; batch 7; 1:500 for IF; 1:2500 for WB); anti-GAPVD1 (Abcam; Cat. ab86033; batch GR265707-8; 1:5000 for WB); anti-VPS4B (Proteintech; Cat. 17673-1-AP; batch 00009255; 1:2500 for WB); anti-exportin-1 (Bethyl Laboratories; Cat. A300-469A; batch 2). Goat: anti-GST (GE Healthcare - Cytiva; Cat. 27457701V; batch 9528344; 1:10,000 for WB); and anti-HA (GenScript; Cat. A00168; batch A102608; 1:2500 for WB; 1:100 for IF). Fluorescent secondary antibodies for microscopy and western blotting were from Jackson ImmunoResearch Laboratories (PA, USA): Alexa Fluor 680-conjugated AffiniPure donkey anti-rabbit IgG (Cat. 711-625-152; batch 139907; 1:5000 for WB); Alexa Fluor 680-conjugated AffiniPure donkey anti-mouse IgG (Cat. 715-625-150; batch 145146/153931; 1:5000 for WB); Alexa Fluor 790-conjugated AffiniPure donkey anti-rabbit IgG (Cat. 711-655-152; batch 132235; 1:5000 for WB); Alexa Fluor 790-conjugated AffiniPure donkey anti-goat IgG (Cat. 705-655-147; batch 143129; 1:5000 for WB); Alexa Fluor 680-conjugated AffiniPure donkey anti-rabbit IgG (Cat. 711-625-152; batch 149402; 1:5000 for WB); Alexa Fluor 680-conjugated AffiniPure donkey anti-goat IgG (Cat. 705-625-147; batch 133233; 1:5000 for WB); Alexa Fluor 488-conjugated AffiniPure donkey anti-mouse IgG (Cat. 715-545-151; 1:800 for IF); Alexa Fluor 594-conjugated AffiniPure donkey anti-mouse IgG (Cat. 715-585-151; 1:800 for IF); Alexa Fluor 488-conjugated AffiniPure donkey anti-rabbit IgG (Cat. 711-545-152; 1:800 for IF); and Alexa Fluor 594-conjugated AffiniPure donkey anti-rabbit IgG (Cat. 711-585-152; 1:800 for IF). TAT1 anti-tubulin was a gift from Keith Gull, University of Oxford [1:2500 for IF (Stefani et al., 2011)]. Custom rabbit anti-Alix (Eurogentec) was generated by using the peptide TPAPRTMPPTKPQPP and affinity purified.

#### Oligonucleotides

For siRNA, the HD-PTP (Doyotte et al., 2008, 2005), Hrs (Flores-Rodriguez et al., 2015), ALIX (Doyotte et al., 2008, 2005), UBAP1 (Stefani et al., 2011) VPS4A and VPS4B (Stefani et al., 2011; Willan et al., 2019) oligonucleotides have been described previously. All siRNA oligonucleotides are listed in Table S1. Allstars negative control siRNA (Qiagen) was used for non-silencing conditions.

#### DNA

##### Mammalian expression vectors

The full-length and Bro1CC truncations of HA-tagged human HD-PTP have been described previously (Doyotte et al., 2008; Gahloth et al., 2017). For FLIM-FRET experiments, human Rab5a was subcloned into NowGFP (Addgene #74749) using the following primers: 5′-GATCGGAAGCTTCTATGGCTAGTC-3′, 5′-GATCGGGGTACCTTAGTTACTAC-3′ and a previously established Rab5-binding domain of human EEA1 was subcloned into mRuby2.C1 (Addgene #54768) using the following primers: 5′-GATCATGTCGACATGATAGAAAAGCTTC-3′, 5′-GATCATCCCGGGTTATCCTTGCAAG-3′.

GFP-tagged human Rabaptin-5 constructs were all subcloned into pEGFP.C1 (Clontech) using standard procedures. The following truncation/ deletion mutants were generated using the following primers: ΔR4BD, 5′-pho-CTGGAGCAGGAGCGAACACAG-3′ and 5′pho-CGGCTGCGCCATGGTACCGTC-3′; ΔR5BD, 5′-GAAAGAGACTGCTGCTTAGGCTACCGTTGAAC-3′ and 5′-GTTCAACGGTAGCCTAAGCAGCAGTCTCTTTC-3′; ΔCC2-1, 5′-pho-TTACAGCAAGCAGAAGACTTCATC-3′ and 5′-pho-CTCCTGAATCTGAATTCCTTGTAAC-3′; ΔCC2-2, 5′-pho-GATGTCAGTGAGCAAGTCCAGAGG-3′ and 5′-pho-CACATGCAGGCTGTGCTTTCCCTG-3′; CC2-1, 5′-ATCGGGTCGACGGTAGACGTTGTGATATGTGTTCCAATTACG-3′ and 5′-GATCATGCGGCCGCCTACAGTGCCTCTGTAGTGTCTGGG-3′; and CC2-2, 5′-GATCGGGTCGACTCATTACAGCAAGCAGAAGACTTCATC-3′ and 5′-GATCATGCGGCCGCCTAGACATCTAATTCTGTCTGTAATCTCTG-3′.

To generate FKBP-HD-PTPMyc, the FKBP fragment was inserted into pcDNA5-HD-PTPmyc (Ali et al., 2013). Mito-FRB was a kind gift from Stephen Royle (University of Warwick, Warwick, UK).

##### Bacterial expression constructs

GST-tagged human Rabaptin-5 constructs were all subcloned into pGEX4T2 (GE Healthcare) using the following primers: CC2-1, 5′-GATCGGGTCGACGGTAGACGTTGTGATATGTGTTCCAATTACG-3′ and 5′-GATCATGCGGCCGCCTACAGTGCCTCTGTAGTGTCTGGG-3′; and CC2-2, 5′-GATCGGGTCGACTCATTACAGCAAGCAGAAGACTTCATC-3′ and 5′-GATCATGCGGCCGCCTAGACATCTAATTCTGTCTGTAATCTCTG-3′. His6–HD-PTP-Bro1CC (including HD-PTP point mutations) were expressed as described previously (Doyotte et al., 2008; Stefani et al., 2011).

##### Yeast two-hybrid constructs

The HD-PTP and Alix constructs (including point mutation variants) were cloned into the EcoRI site of pGBKT7 bait, using the Clontech In-Fusion method, as previously described (Ali et al., 2013; Stefani et al., 2011). Rabaptin-5 was cloned into pGADT7 using the Clontech In-Fusion method.

### Cell culture, transfections and treatments

HeLa (ATCC CCL-2) and HEK293T (ATCC CRL-3216) cells were grown in Dulbecco's modified Eagle's medium (DMEM) supplemented with 1% non-essential amino acids (NEAA, Sigma), 10% fetal calf serum (HyClone; Perbio) and 1% glutamine (Sigma) at 37°C and 8% CO_2_. Cells were verified and checked routinely for contamination. HeLa DNA transfections were performed using Fugene 6 (Roche), GeneJuice (Millipore) or Lipofectamine 2000 (Invitrogen). HEK293T DNA transfections were performed using Branched PEI Mw - 25 kDa (Sigma): briefly, complexes of DNA:PEI (1 mg/ml) at a ratio of 1:3 were formed and cells were analysed at 20–24 h post transfection. Transfection with siRNA duplexes (Table S1) was performed using INTERFERin (Polyplus) according to the manufacturer's instructions. HB-EGF (Sigma) was used at 100 ng/ml in Optimem (Gibco) containing 0.2% bovine serum albumin (BSA). Cells were treated with 30 µM CID755673 (Generon) in Optimem for up to 6 h. For the mitochondrial relocation assay, cells were treated with 1 µM rapamycin for 3 h. When siHD-PTP-treated cells were additionally transfected with a plasmid DNA during the knockdown, the DNA transfection was carried out 28–32 h after the siRNA transfection, and the cells were incubated for an additional 16-20 h.

### Yeast two hybrid

Yeast two-hybrid screening was performed by Hybrigenics, S.A., Paris, France (http://www.hybrigenics-services.com). Interactions were further tested using the Clontech ‘Matchmaker Gold’ system (Clontech), as described previously (Ali et al., 2013; Stefani et al., 2011). Briefly, test plasmids were co-transformed into the yeast strain Y2HGold and grown on minimal medium agar plates (double drop-out; DDO), deficient in tryptophan and leucine, to select for the presence of the bait (pGBKT7) and prey (pGADT7) plasmids, respectively. Doubly transformed colonies were then streaked onto minimal quadruple drop-out (QDO) plates, additionally deficient in histidine and adenine to select for bait and prey interactions. Agar, yeast nitrogen base without amino acids and amino acid mixes were from Formedium.

### EGFR internalisation analysis

HeLa cells were serum starved for 6 h at 37°C and incubated with anti-EGFR mAb 108 for 1 h on ice to achieve labelling of the cell surface population of the receptor. After washing with ice-cold PBS to remove any unbound antibody, cells were incubated with 100 ng/ml HB-EGF (R&D Systems) in L15/BSA (Leibovitz's medium containing 0.2% BSA) for 1 h on ice. Cells were then washed with ice-cold PBS and incubated for 0–180 min at 37°C in pre-warmed, ligand-free L15/BSA to allow receptor internalisation. At the end of the chase period, cells were washed and fixed for immunofluorescence microscopy.

### Immunofluorescence, imaging and analysis

For CHMP4B, EGFR, EEA1, FK2, HA, Rab5, MYC and mtHsp70 labelling, cells were fixed in 4% formaldehyde for 10 min and quenched with 0.1 M glycine, 50 mM Tris-HCl pH 7.5 for 5 min, then permeabilised for 10 min in PBS containing 0.1% Triton X-100. For mouse anti-Hrs and rabbit anti-HD-PTP staining, cells were fixed in methanol at −20°C. Standard fluorescence experiments were imaged on an Olympus BX51 upright microscope fitted with a 60×1.4 NA Plan Apo objective and CoolSnap ES camera, and 12-bit images captured using MetaVue software. For FLIM-FRET experiments, image acquisition and analysis was performed on a Leica SP8 FALCON inverted confocal laser scanning microscope. Lifetime images were generated on FLIMfit software (Warren et al., 2013). For the mitochondrial targeting assay, following 16 h DNA transfection, cells were treated with 1 µM rapamycin (Millipore) for 3 h at 37°C. Cells were then formaldehyde fixed and stained.

All images were processed and analysed using FiJi. To quantify the percentage of normal cells, the proportion of normal cells in 9 randomly selected regions of interest was calculated by researchers who were blind to the experimental conditions; any cells that did not exhibit enlarged or clustered endosomal morphology were considered normal. For endosomal size measurements, images were thresholded using the Moments algorithm (Tsai, 1985) against the EEA1 or Rab5 channel to detect endosomal edges and the surface area of these structures was measured.

Colocalisation of markers was assessed using a non-biased, object-based technique. First, random views of cells were imaged and a grid was imposed using ImageJ. To achieve random sampling, each EGFR-positive endosome that intersected with a horizontal line was selected and then assessed for whether it also labelled for the given maker protein.

### Electron microscopy

For EM pulse-chase analysis, 18 nm colloidal gold particles were conjugated to affinity-purified anti-EGFR antibody Mab 108. Cells were incubated with gold conjugates in serum-free DMEM buffered with 20 mM HEPES and containing 1% (w/v) BSA (binding medium) for 90 min at 4°C in order to allow antibody binding to surface EGFR (Futter et al., 2001). Cells were then washed and incubated at 37°C for a specified time in fresh binding medium containing 100 ng/ml HB-EGF to stimulate the internalisation of EGFR, before being fixed and processed for EM. Cells in culture were trypsinised and then fixed by adding an equal volume of 4% paraformaldehyde, 0.1% glutaraldehyde in 0.1 M calcodylate buffer, pH 7.4, containing 3 µM CaCl_2_ 7.5% (w/v) sucrose and were incubated for 15 min at 37°C. Fixed cells were washed with 0.1 M calcodylate buffer before being pelleted (500 ***g*** for 10 min). The cell pellet was stained in reduced osmium tetraoxide [a 1:1 mixture of 2% OsO_4_ (TAAB, UK) in H_2_O and 3% Fe_3_(CN)_6_ in 0.1 M calcodylate] for ∼60 min. Samples were dehydrated with an ethanol series, then transferred into propylene oxide (TAAB, UK) and then a 1:1 mixture of propylene oxide and resin, for resin infiltration. Following more than 120 min of infiltration, the mixture was replaced with pure resin. After at least 6 h, the resin was replaced with fresh resin and this was polymerised in an 85°C oven for 24 h. Ultrathin serial sections were stained with 0.3% lead citrate and observed with an FEI Tecnai 12 Biotwin at 100 kV. Images were taken using an integrated Orius SC1000 (model 832) Gatan CCD camera (Gatan Inc., USA) using DigitalMicrograph software (Gatan Inc.).

### Subcellular fractionation

Trypsinised HeLa cells (10^7^) were collected in 50 ml complete DMEM and washed twice with ice-cold buffer A (10 mM HEPES pH 7.4, 3 mM magnesium acetate, 5 mM EGTA, 250 mM sucrose). Cells were then treated with 1 ml buffer A supplemented with protease inhibitor cocktail and 1 mM DTT on ice for 10 min before being homogenized by passing 8–10 times through a ball-bearing homogenizer (Isobiotec, Germany). Homogenised cells were subjected to two rounds of centrifugation at 700 ***g*** for 10 min at 4°C to remove unbroken cells and nuclei. The post-nuclear supernatant was loaded on top of an ice-cold sucrose cushion [25% sucrose (w/v) in buffer A] and centrifuged in a TLS-55 rotor at 100,000 ***g*** for 30 min at 4°C to sediment total cellular membranes. The cytosol was removed carefully and the pelleted membranes were washed with buffer A, pelleted (100,000 ***g*** for 30 min at 4°C) and solubilised directly into SDS-PAGE sample buffer.

### Protein extraction and western blotting

Cells were solubilised on ice in immunoprecipitation (IP) buffer [25 mM Tris-HCl pH 7.5, 150 mM NaCl, 0.5 mM EDTA, 0.5% (v/v) IGEPAL CA-630] supplemented with 2 mM Na_3_VO_4_, 50 mM β-glycerophosphate, 50 mM NaF, 10 mM NEM, 1 mM PMSF and protease and phosphatase inhibitor cocktails [all reagents form Sigma, expect for Tris, NaCl and EDTA (Thermo Fisher Scientific), P2 and P3 phosphatase inhibitor (Merck P5726 and P0044) and protease inhibitor (Merck 4693132001)] by shaking at 4°C for 1 h. Detergent-insoluble material was cleared by centrifugation at 17,000 ***g*** for 10 min at 4°C. For CIP treatment, post-nuclear lysates prepared as described above were treated with CIP (New England Biolabs; 0.5 U/µl final) for 1 h at 37°C.

To perform western blotting of total cell extracts, cells in dishes were washed three times in PBS, and then lysed in 2× SDS-PAGE sample buffer and boiled (Rabaptin-5 hyperphosphorylation samples were gently heated at 37°C for 1 h). Samples were run on polyacrylamide gels and transferred to PVDF membranes (Millipore). Blots were probed with IRDye 680 CW and IRDye 800 CW secondary antibodies (Li-Cor Bioscience) and scanned with a Li-Cor Bioscience Odyssey scanner. Quantitation was performed using ImageJ software.

### Coimmunoprecipitations

For native IPs, cells were lysed in IP buffer [25 mM Tris-HCl pH 7.5, 150 mM NaCl, 0.5% (v/v) IGEPAL CA-630, 1× phosphatase inhibitor cocktail 2 and 3 (Sigma), 1× protease inhibitor cocktail EDTA-free tablet (Roche)]. Lysates were clarified at 10,000 ***g*** for 30 min at 4°C and incubated with antibodies at 4°C for at least 4 h, then 1-2 h with 10 µl protein A or protein G Sepharose slurry (Zymed), pre-blocked with 50 mg/ml BSA. Beads were washed three times in IP buffer, then analysed by western blotting (WB). Control IPs used IgGs. Panels for all figures have been cut from the same exposures unless stipulated in the figure legend.

### Recombinant protein production and pulldown assays

Recombinant proteins were produced in BL21 (DE3) *E.coli* via 0.5 M IPTG induction for 3 h at 30°C. Following *E. coli* sonication, protein was purified on glutathione agarose (GE Healthcare) or NTA (Invitrogen) beads, eluted, snap frozen and stored until use. 0.4 mg GST or GST-tagged Rabaptin-5 was incubated with 10 µl glutathione agarose bead slurry in HTG buffer [50 mM HEPES pH 7.4, 150 mM NaCl, 0.1% Triton X-100 (Anatrace), 10% glycerol] for 1 h at 4°C with rotation. Beads were washed three times in HTG buffer and 2 µg His_6_–HD-PTP-Bro1CC was added and incubated at 4°C for 3 h. Beads were washed three times in IP buffer, then analysed by western blotting. WT or L217A CHMP4B small peptides (amino acids 205–224) were produced by Generon Ltd.

### Experimental design and statistical analysis

All data are presented as mean±s.d. unless otherwise stated. Data were first tested for normality using the D'Agostino-Pearson omnibus K2 normality test to determine the appropriate statistical test. Since all data with large sample size were found to fit a normal distribution, parametric statistics were employed. For experiments with *n* numbers lower than 8, data was assumed to fit a normal distribution without formal testing. Unpaired two-tailed Student's *t*-tests were used for statistical comparisons between two groups, and for experiments containing three or more groups, one-way analysis of variance (ANOVA) was employed. Two-way ANOVAs were used to analyse data affected by two categorical independent variables. To compare all variables with one another, a Bonferroni's post-hoc test for multiple comparisons was used. All statistical tests were performed on GraphPad Prism (v6.0) and differences were considered significant if *P*<0.05 (denoted *P<0.05, **P<0.01, ***P<0.001).

## Supplementary Material

Supplementary information

Reviewer comments
