# Supplementary material for: His domain protein tyrosine phosphatase and Rabaptin-5 couple endo-lysosomal sorting of EGFR with endosomal maturation
Source: J Cell Sci. 2021 Nov 4;134(21):jcs259192. doi: 10.1242/jcs.259192 (PMC8627557; doi:10.1242/jcs.259192)
Supplement: Supplementary information [file joces-134-259192-s1.pdf]

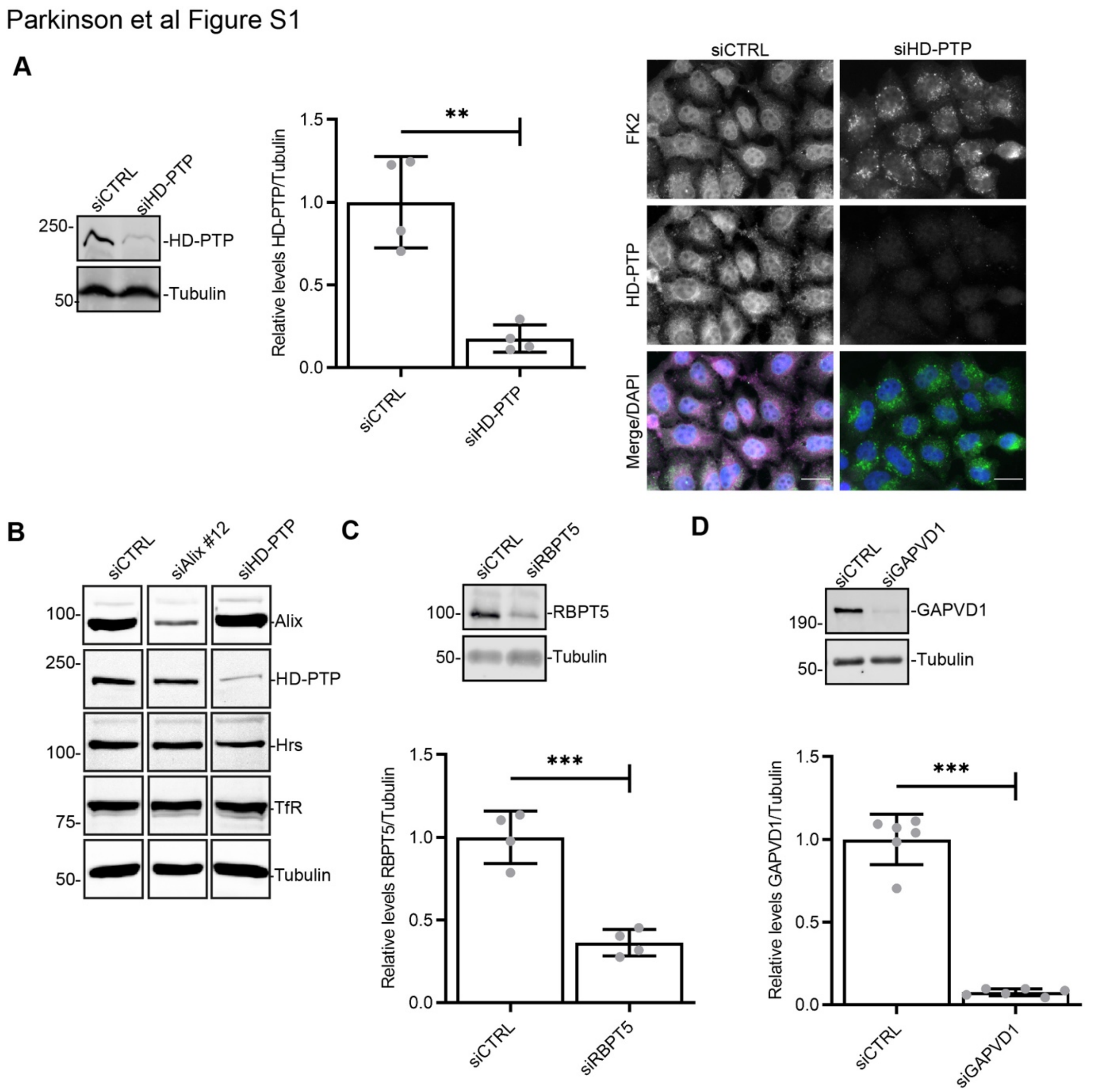

Fig. S1. A) Validation of siRNA silencing of HD-PTP. Left: lysates from HeLa cells were harvested and proteins detected by Western blotting. Graph shows quantification of total protein expression relative to tubulin, values represent mean ± SD. Unpaired two-tailed student's t-test, $^{**}p<0.01$, n=4. Right: HeLa cells were immunostained for HD-PTP (red) and with FK2 anti-ubiquitin conjugate (green). Scale bar = 10 µm. B) Validation of siRNA silencing of Alix. C) Validation of siRNA silencing of Rabaptin-5. Unpaired two-tailed student's t-test, $^{***}p<0.001$, n=4. D) Validation of siRNA silencing of GAPVD1. Unpaired two-tailed student's t-test, $^{***}p<0.0001$, n=6.

Journal of Cell Science • Supplementary information

Parkinson et al Figure S2

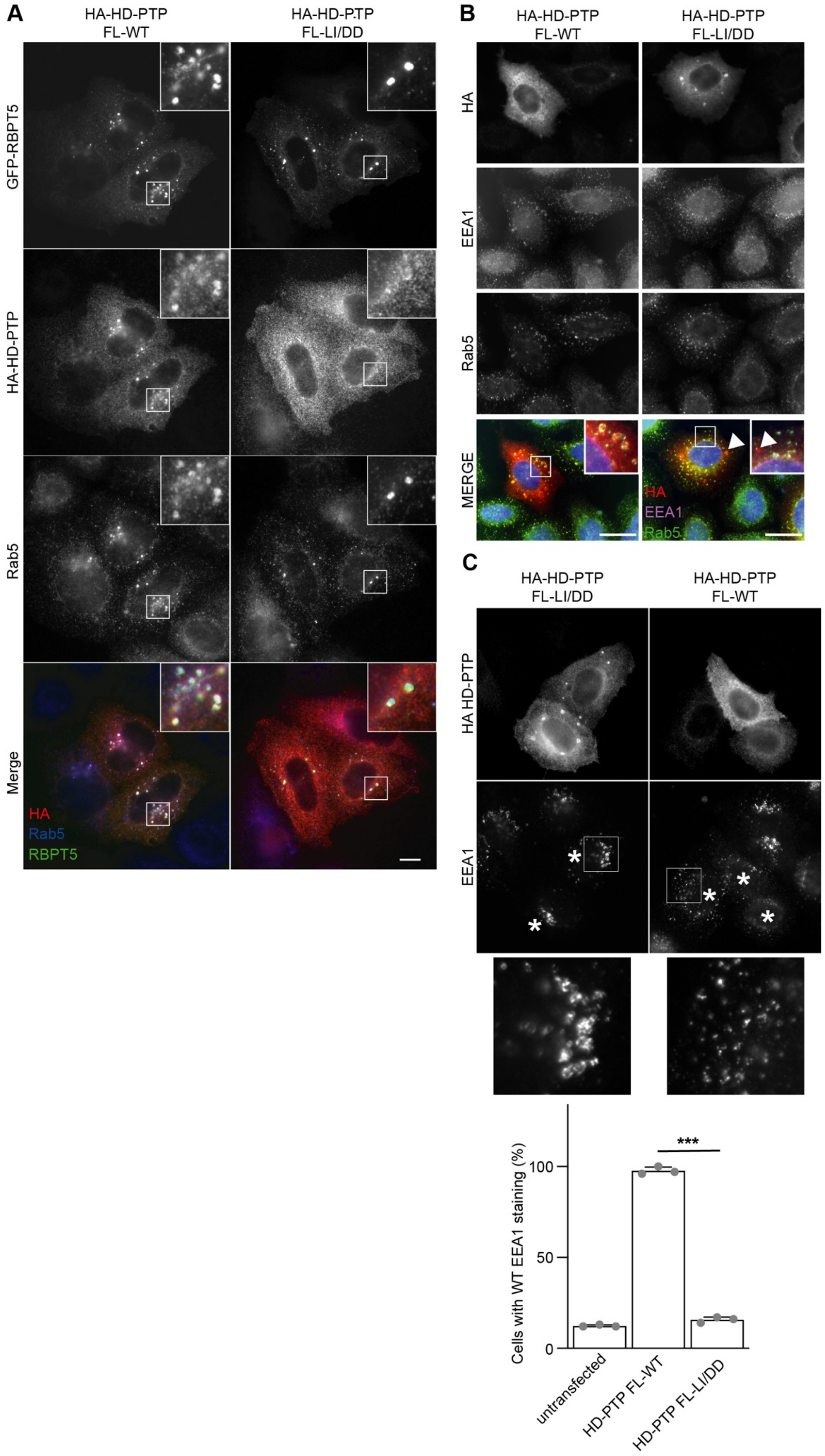

Journal of Cell Science • Supplementary information

Fig. S2. A) HeLa cells were transfected with HA-HD-PTP and GFP-Rabaptin-5 constructs as
indicated (FL = full-length), and labelled for endogenous Rab5. Scale bar represents 10 µm. Insets magnified x 3.
B) HeLa cells were transfected with HA-HD-PTP constructs and labelled as indicated. Arrowheads indicate non-endosomal HA-HD-PTP clusters. Scale bar represents 20 µm. Insets magnified x 3.
C) Left: HeLa cells were depleted by siRNA and rescued with RNAi-resistant HA-HDPTP as indicated and immunostained for EEA1. HA-HD-PTP transfected cells are asterisked. Scale bar represents 10 µm. Insets magnified x4. Right: Histogram showing the percentage of cells that displayed a normal distribution of EEA1 (mean ± SD from 3 independent experiments, 100 cells counted per experiment). One-way ANOVA with Bonferroni's test for multiple comparisons, ***p=0.0004.

Table S1. siRNA oligonucleotides used in this study.

Protein	Company	Commercial name	Sequence	Conditions
HD-PTP	Thermo Scientific	J-009417-06	GCAAACAGCGGAUGAGCAA	5nM, 48hr
Rabaptin-5	Qiagen	Custom	GUAGUAUGCUGUAUGAAUA	10nM, 48hr
ALIX	Thermo Scientific	J-004233-12	GUACCUCAGUCUAUAUUGAUU	20nM, 48hr followed by 20nM, 48hr
GAPVD1	Thermo Fisher Scientific	s25136		5nM, 48hr
UBAP1	Qiagen	Custom	CCCAAUGGCUUUAUAACCUUA	20nM, 72hr
Hrs	Thermo Scientific	J-016835-06	GCACGUCUUUCCAGAAUUC	20nM, 72hr
VPS4A	Thermo Scientific	L-013092-05	CCACAAACAUCCCAUGGGU	5nM, 72hr
VPS4B	Thermo Scientific	L-013119-05	GGGCAAAGUGUACAGAAUA	5nM, 72hr

Journal of Cell Science • Supplementary information
