# Supplementary material for: His domain protein tyrosine phosphatase and Rabaptin-5 couple endo-lysosomal sorting of EGFR with endosomal maturation
Source: J Cell Sci. 2021 Nov 4;134(21):jcs259192. doi: 10.1242/jcs.259192 (PMC8627557; doi:10.1242/jcs.259192)
Supplement: Reviewer comments [file joces-134-259192_review_history.pdf]

His domain protein tyrosine phosphatase and Rabaptin-5 couple endo-lysosomal sorting of EGFR with endosomal maturation

Gabrielle Parkinson, Peristera Roboti, Ling Zhang, Sandra Taylor and Philip Woodman
DOI: 10.1242/jcs.259192

Editor: Giampietro Schiavo

Review timeline

Original submission:	26 July 2021
Editorial decision:	16 August 2021
First revision received:	7 September 2021
Accepted:	13 September 2021

Original submission

First decision letter

MS ID#: JOCES/2021/259192

MS TITLE: His Domain Protein Tyrosine Phosphatase and Rabaptin-5 couple endo-lysosomal sorting of EGFR with endosomal maturation

AUTHORS: Gabrielle Parkinson, Peristera Roboti, Ling Zhang, Sandra Taylor, and Philip Woodman
ARTICLE TYPE: Research Article

We have now reached a decision on the above manuscript.

To see the reviewers' reports and a copy of this decision letter, please go to: https://submit-jcs.biologists.org and click on the 'Manuscripts with Decisions' queue in the Author Area. (Corresponding author only has access to reviews.)

As you will see, the reviewers gave favourable reports on your manuscript but raised some critical points that will require amendments. I hope that you will be able to carry these out in the next couple of weeks, because I would like to be able to accept your paper, depending on further comments from reviewers.

We are aware that you may be experiencing disruption to the normal running of your lab that makes experimental revisions challenging. If it would be helpful, we encourage you to contact us to discuss your revision in greater detail. Please send us a point-by-point response indicating where you are able to address concerns raised (either experimentally or by changes to the text) and where you will not be able to do so within the normal timeframe of a revision. We will then provide further guidance. Please also note that we are happy to extend revision timeframes as necessary.

Please ensure that you clearly highlight all changes made in the revised manuscript. Please avoid using 'Tracked changes' in Word files as these are lost in PDF conversion.

I should be grateful if you would also provide a point-by-point response detailing how you have dealt with the points raised by the reviewers in the 'Response to Reviewers' box. Please attend to all of the reviewers' comments. If you do not agree with any of their criticisms or suggestions please explain clearly why this is so.

© 2021. Published by The Company of Biologists under the terms of the Creative Commons Attribution License (https://creativecommons.org/licenses/by/4.0/).

Reviewer 1

Advance summary and potential significance to field

The paper shows a direct interaction between HD-PTP and Rabaptin. It has previously been reported to be involved in MVB formation. This paper shows that HD-PTP may have an additional role in facilitating endosomal maturation by interfering with a positive feedback loop between Rab5 and it's GEF. I feel the paper is interesting and makes a significant contribution to the field.

Paper is well written, data nicely presented and conclusions well supported by the data.

Comments for the author

I do not feel that there is really anything major that is essential to address. Possibly the only thing is that I would like the authors to comment why the membrane association status of Rabaptin 5 is not shown in Fig 4A.

Most of my comments are minor. See below.
Results Depletion of HD-PTP results in accumulation of EGFR in endosomal/Rab5 positive compartment as shown in Figure 1 and 2.
Data is convincing although in Fig1A (30min HB-EGF stimulation) there appeared much more EGFR (gold particles) in the control than in siHD-PTP.
Seems slightly at odds with the fluorescence microscopy where there appears EGFR concentrated in small areas in knockdown cells. Not a major criticism.
More an observation. It would have been good to have the prevention of EGFR degradation in knockdown cells confirmed by immunoblotting (maybe this has been done in a previous paper and can be commented on in the results) and also the ubiquitylation status of EGFR in the knockdown cells addressed, but this may not be technically feasible.

In figure 3 evidence is presented to show that active Rab5 accumulates on endosomes. I felt this data is solid although I am not so familiar with fluorescence lifetime measurements. Possibly include a sentence explaining how decreased fluorescence lifetime equates to increased association of Rab5 and effector. The quantification graphs in panels A and B are a little subjective, requiring judgement of the researcher whether a cell looks normal or not, but there are clear differences in appearance of control and knockdown cells so not a major issue.

It is interesting that co-knockdown of Rabaptin5 seems to rescue the HD-PTP knockdown somewhat. It made me wonder whether the co-knockdown had been tried in HB-EGF stimulated cells. If so, what is the fate of EGFR? I would assume it still does not get sorted into MVB's but does EGFR progress to a Rab7 compartment?
Figure 4. Shows that phosphorylated Rabaptin accumulates upon HD-PTP depletion and that phosphorylation could be by PKD. Again the data seems good and supports these conclusions. In panel A it is shown that Rabex5 membrane association is not altered by HD-PTP knockdown. The membrane association of Rabaptin5 is however not shown. I would have thought that this was quite an obvious thing to check. Was this done? I feel that this is quite important and could at least be commented upon in the discussion. Is phosphorylation of Rabaptin5 affecting its interaction with Rabex5/membranes and GEF activity?
Figure 5 and 6 show the interaction between HD-PTP and map the interacting regions. Data support the conclusions.

Minor comments. Line 285. The way it is written made me think that HEK293T cells were used for expression because they do not express endogenous Rabaptin-5 or low levels. Is this the case?
Discussion As far as I understand the proposed mechanism is that binding of HD-PTP to Rabaptin 5 is switching of positive feedback loop of GEF/Rab5 activation.
So, without HD-PTP positive feedback loop between RAB and GEF is not disrupted. In the normal situation it is not clear to me what the driver for switching from ESCRT-III binding to Rabaptin5 binding could be. How is 'completion of cargo sorting' sensed? I felt there could be a bit more speculation about the potential mechanism, possibly with an altered Figure 7 in the discussion.

© 2021. Published by The Company of Biologists under the terms of the Creative Commons Attribution License (https://creativecommons.org/licenses/by/4.0/).

Reviewer 2

Advance summary and potential significance to field

The authors have shown a direct interaction between HD-PTP and Rabaptin-5, and investigated the role of HD-PTP in endosomal function. This builds on work from this group and others, and leads to a new model for HD-PTP function in coordinating endosomal maturation with MVB sorting.

Comments for the author

This is a well written paper, and includes good biochemical protein interaction data. There are some areas where tidying up is required, some figures could be more clearly labelled and explained, and some statements that need aligning more to the data as shown.

1. Please define HB-EGF (heparin-binding EGF-like growth factor), line 120.

2. Why are there two bands for tubulin in Fig. S1C, when all other gels show a single band? Are the protein samples degraded?

3. The images shown in Fig. 1B show an accumulation of EGFR in EEA1+ endosomes when HD-PTP is depleted. This is by eye, from the images shown. Quantitation of this would strengthen the argument considerably. Similarly, the images in Fig. 2 require quantitation of colocalization, and the arrows need shifting to point directly to the puncta (the top row is missing arrows, the second row has arrows that don't appear to line up image- image [the left and middle images specifically] and the arrows seem quite far away from the relevant spots).

4. Fig. S2 is important, and perhaps ought to be included in the main paper if space allows. How exactly was endosome area calculated from these images? Are other phenotypes of HD-PTP depletion also rescued (e.g. the EGFR internalization after HB-EGF stimulation)?

5. In Fig. 4D, there does indeed appear to be some reduction in the higher MW forms of Rabaptin 5 after CIP treatment, but "substantially reduced" (lines 226-227) may be overstating it a little. The graph in 4E shows the reduction in higher MW forms (in response to a more specific inhibitor) more clearly, but what happens at 6h? This needs a comment.

6. I'm afraid I do not see the "modest increase in GFP-Rabaptin-5 phosphorylation upon co-transfecting with mApple-Rab4" or the "strongly enhanced GFP-Rabaptin-5 phosphorylation in HD-PTP-depleted cells" in Fig. 4G (lines 244-248). The bands are closer together than in previous blots due to the increased molecular weight of GFP-Rabaptin-5 over untagged, but this makes them difficult to distinguish. I don't think these conclusions can be drawn from these data. Better separation of phosphorylated from unphosphorylated GFP-Rabaptin-5 is required.

7. Fig. 5A. Please label HD-PTP and Rabaptin-5. Presumably the spot and the arrow represent the L202 important for CHMP4 binding? Labelling this would help the reader follow later points in the paper.

8. In Fig. 5B, which contructs are transfected (in addition to HD-PTP constructs, presumably Rabaptin-5 – full length or 630-862?) and on which medium are the yeast grown? Presumably one would expect growth on this medium to indicate interaction? More details are needed, especially since none are given (other than reference to a kit) in the methods. Many panels are blank in this figure, which tells us little. One would normally show yeast growing on selective plates, and then replicate them onto plates to show growth only with expression of the reporter. This shows all yeast have the appropriate plasmids transformed (and are growing!) and shows which constructs can interact. Please include this. What is the relevance of HD-PTP Bro1-CC (F678), as I can find no other reference to it in the manuscript? Please either explain or remove.

9. In Fig. S3, what are HA-HD-PTP-FL-WT and HA-HD-PTP-FL-LI/DD (what is "FL"?)? I assume the cells are transfected with GFP-RBPT5 and an HA-tagged HD-PTP (WT or the LI/DD). It would be clearer to state what the cells were transfected with at the top and then label the panels going down with GFP, HA, Rab5.

© 2021. Published by The Company of Biologists under the terms of the Creative Commons Attribution License (https://creativecommons.org/licenses/by/4.0/).

10. In Fig. 5F, how do lanes 1 and 2, and 4 and 5 differ? The legend does not explain, but it looks as though GFP alone has been used as a control.

11. Fig. 6A and B. While I agree that in Fig. 6A less CC2-2 seems to be pulled down with HD-PTP-Bro1-CC with the CHMP4B-binding pocket mutated (lines 308-312), in the gel in Fig. 6B it looks as though the same is true for CC2-1. I see no band at all in the figure, though a band is clear for the pull down of CC2-1 with WT-HD-PTP. The graph suggests otherwise. Is the gel not a particularly good example here? I assume the graph uses averages of several experiments, so another gel should be substituted.

12. Fig. 6C. What does the sh stand for in e.g. WT CHMP4B.sh?

13. Fig. 6D. Do all these mutations affect endofin interaction? I think more than one sentence is needed to explain this figure (lines 328-329). Why do some mutations have more effect on Rabaptin-5 binding than others?

Reviewer 3

Advance summary and potential significance to field

The manuscript is of general interest to the Cell Biology community for the following two reasons:

1. The conversion of Rab5 to Rab7 endosomes induces MVB maturation into a lysosome fusion-competent state. This switch is an important step in ensuring the efficient transport of endocytosed proteins and an important step in cellular membrane quality control. How Rab conversion occurs concomitantly with MVB formation is unknown. The authors in this paper propose a model that HDPTP an ESCRT- protein, is a central player in integrating Rab conversion with ESCRT-mediated MVB formation.

2. HDPTP and ALIX are two similar proteins. The role of ALIX in ESCRT-related processes has been studied quite extensively. However, HDPTP has not been studied to the same extent. The authors here provide a compelling argument for HDPTP's role in endosomal maturation.

In the manuscript, the authors demonstrate that HDPTP is involved in interacting with Rabaptin-5, while Alix does not, and this interaction may facilitate endosome maturation. The authors also determine the binding site of Rabaptin to HDPTP. Overall, the paper is very well written, consist of a new finding, the data are well controlled, and the model is appropriate with the data presented.

Comments for the author

I only have minor suggestions, regardless of which the paper is suitable for publication, and will be interest to the ESCRT and endosome/membrane-trafficking communities.

a. The title is somewhat misleading to a reader not familiar with HDPTP.
Since the work is about an increases in phosphorylation of Rabaptin upon HDPTP inhibition, a reader may think that HDPTP is the phosphatase. A mention of this in the abstract (saying HDPTP is probably not be the phosphatase) maybe better.
Otherwise the authors would have to perform in-vitro assays to demonstrate phosphatase activity of HDPTP.

b. Related to Fig 2: in the text, it is not mentioned that Alix knockdown does have an effect in MVB sorting. This should be mentioned explicitly, unless the authors are suggesting that Alix is not important for MVB sorting, which would be surprising to me. Fig. 2 does show punctate structures that are supposed to be un-degraded EGFR.

c. HD-PTP binds Rabaptin at the CHMP4-binding site. The specificity of this interaction is provided by the "S-pocket" in HDPTP, which is not present in Alix. Some sequence/structural

© 2021. Published by The Company of Biologists under the terms of the Creative Commons Attribution License (https://creativecommons.org/licenses/by/4.0/).

information about this pocket as an accompanying figure in Fig. 6 would be helpful for the readers, to demonstrate this interesting difference between HDPTP and Alix.

d. In the Discussion, line 345, I'd suggest replacing the word "control" with a less strong word. While I agree from the data that HDPTP binds to Rabaptin and is involved in facilitating endosomal maturation with MVB sorting I'm not sure we can say that HDPTP controls endosome maturation.

First revision

Author response to reviewers' comments

Reviewer 1
It would have been good to have the prevention of EGFR degradation in knockdown cells confirmed by immunoblotting (maybe this has been done in a previous paper and can be commented on in the results) and also the ubiquitylation status of EGFR in the knockdown cells addressed, but this may not be technically feasible.

- As the reviewer suggests, these data have been presented in previous papers, to confirm that HD-PTP knockdown prevents the deubiquitination and degradation of EGFR (assessed by immunoblotting). We now refer to these studies in the Results. Lines 142-145.
In figure 3 evidence is presented to show that active Rab5 accumulates on endosomes. I felt this data is solid although I am not so familiar with fluorescence lifetime measurements. Possibly include a sentence explaining how decreased fluorescence lifetime equates to increased association of Rab5 and effector.

- A sentence has been included to explain briefly the principles of FRET and FLIM. Lines 200-202.
The quantification graphs in panels A and B are a little subjective, requiring judgement of the researcher whether a cell looks normal or not, but there are clear differences in appearance of control and knockdown cells so not a major issue.

- We have included in the Methods section clarification as to how phenotypes were categorized. Lines 621-624.
It is interesting that co-knockdown of Rabaptin5 seems to rescue the HD-PTP knockdown somewhat. It made me wonder whether the co-knockdown had been tried in HB-EGF stimulated cells. If so, what is the fate of EGFR? I would assume it still does not get sorted into MVB's but does EGFR progress to a Rab7 compartment?

- This is an interesting suggestion, but we feel this goes outside the remit of the paper. The experiment would be better attempted when we know precisely how Rabaptin and HD-PTP interact, and thus we can use non-binding mutants.
Figure 4. Shows that phosphorylated Rabaptin accumulates upon HD-PTP depletion and that phosphorylation could be by PKD. Again the data seems good and supports these conclusions. In panel A it is shown that Rabex5 membrane association is not altered by HD-PTP knockdown. The membrane association of Rabaptin5 is however not shown. I would have thought that this was quite an obvious thing to check. Was this done? I feel that this is quite important and could at least be commented upon in the discussion. Is phosphorylation of Rabaptin5 affecting its interaction with Rabex5/membranes and GEF activity?

- We have included an additional experiment (new Figure 5F) to test the association of Rabaptin-5 with membranes upon HD-PTP depletion. As for Rabex-5, the membrane association of Rabaptin-5 is unaltered. This suggests that HD-PTP influences the Rab5 GEF catalytic cycle, rather than simply preventing the GEF complex from assembling on the membrane, but further studies are required to understand the mechanism here. New text has been added. Lines 241-245.
Figure 5 and 6 show the interaction between HD-PTP and map the interacting regions. Data support the conclusions.Minor comments. Line 285. The way it is written made me think that HEK293T cells

© 2021. Published by The Company of Biologists under the terms of the Creative Commons Attribution License (https://creativecommons.org/licenses/by/4.0/).

were used for expression because they do not express endogenous Rabaptin-5 or low levels. Is this the case?

- We have altered the phrasing here, to make the intention clearer. Lines 279-284.

Discussion

As far as I understand the proposed mechanism is that binding of HD-PTP to Rabaptin 5 is switching of positive feedback loop of GEF/Rab5 activation. So, without HD-PTP positive feedback loop between RAB and GEF is not disrupted. In the normal situation it is not clear to me what the driver for switching from ESCRT-III binding to Rabaptin5 binding could be. How is ‘completion of cargo sorting’ sensed? I felt there could be a bit more speculation about the potential mechanism, possibly with an altered Figure 7 in the discussion.

- We have modified Figure 7 (now Figure 8) and figure legend, and changed the text in the discussion to make this point clearer. Lines 414-416 and 421-424.

Referee 2

1. Please define HB-EGF (heparin-binding EGF-like growth factor), line 120.
- Corrected. See lines 130-131.

2. Why are there two bands for tubulin in Fig. S1C, when all other gels show a single band? Are the protein samples degraded?
- We agree that the blot could have been better chosen. Another blot is shown.

3. The images shown in Fig. 1B show an accumulation of EGFR in EEA1+ endosomes when HD-PTP is depleted. This is by eye, from the images shown. Quantitation of this would strengthen the argument considerably. Similarly, the images in Fig. 2 require quantitation of colocalization, and the arrows need shifting to point directly to the puncta (the top row is missing arrows, the second row has arrows that don’t appear to line up image- image [the left and middle images specifically] and the arrows seem quite far away from the relevant spots).
- We have quantified the images as requested, and also made the suggested alterations to Figure 2. Quantification is shown in the image panels. We have included details of the quantification protocol in the methods section. Lines 628-632.

4. Fig. S2 is important, and perhaps ought to be included in the main paper if space allows. How exactly was endosome area calculated from these images? Are other phenotypes of HD-PTP depletion also rescued (e.g. the EGFR internalization after HB-EGF stimulation)?
- We have moved Fig. S2 to Fig. 3 as requested (and altered the forward figure numbers). We have added information in the methods section to show how the endosome area was calculated. Lines 625-627. We have not examined whether other phenotypes are rescued, since the size/clustering of endosomes is the focus of this paper on Rab5 activation. However, our previous work has shown that other phenotypes of HD-PTP depletion (such as aberrant ubiquitin homeostasis on endosomes) are rescued by expression of RNAi resistant HD-PTP (Doyotte et al. 2008).

5. In Fig. 4D, there does indeed appear to be some reduction in the higher MW forms of Rabaptin 5 after CIP treatment, but “substantially reduced” (lines 226-227) may be overstating it a little. The graph in 4E shows the reduction in higher MW forms (in response to a more specific inhibitor) more clearly, but what happens at 6h? This needs a comment.
- We have removed the word “substantially” and modified the text to say “The MW shift is due, at least in part…”. Line 233. At 6 h there is a slight reduction (compared to 4 h) in the sensitivity to the PKD inhibitor, but the effects are still significant and we believe the reader will not need this pointing out.

6. I’m afraid I do not see the “modest increase in GFP-Rabaptin-5 phosphorylation upon co-transfecting with mApple- Rab4” or the “strongly enhanced GFP-Rabaptin-5 phosphorylation in HD-PTP-depleted cells” in Fig. 4G (lines 244-248). The bands are closer together than in previous blots due to the increased molecular weight of GFP-Rabaptin-5 over untagged, but this makes them difficult to distinguish. I don’t think these conclusions can be drawn from these data. Better separation of phosphorylated from unphosphorylated GFP-Rabaptin-5 is required.

© 2021. Published by The Company of Biologists under the terms of the Creative Commons Attribution License (https://creativecommons.org/licenses/by/4.0/).

- We accept that the reviewer finds these effects marginal. Since these experiments are not central to the narrative, we think it simplest to exclude them. Hence, the original Figure 4E/F have been removed, along with the corresponding text.

7. Fig. 5A. Please label HD-PTP and Rabaptin-5. Presumably the spot and the arrow represent the L202 important for CHMP4 binding? Labelling this would help the reader follow later points in the paper.
- We have corrected the figure and figure legend.

8. In Fig. 5B, which contructs are transfected (in addition to HD-PTP constructs, presumably Rabaptin-5 – full length or 630-862?) and on which medium are the yeast grown? Presumably one would expect growth on this medium to indicate interaction? More details are needed, especially since none are given (other than reference to a kit) in the methods. Many panels are blank in this figure, which tells us little. One would normally show yeast growing on selective plates, and then replicate them onto plates to show growth only with expression of the reporter. This shows all yeast have the appropriate plasmids transformed (and are growing!) and shows which constructs can interact. Please include this. What is the relevance of HD-PTP Bro1-CC (F678), as I can find no other reference to it in the manuscript? Please either explain or remove.
- This was an oversight and the reviewer is absolutely correct that this information should have been included. We now show the yeast growth in double dropout (DDO) selection medium (which selects for expression of the constructs) and quadruple dropout (QDO) selection medium, which selects for the presence of the interaction. Notably, the combination of Bro1 domain and Rabaptin-5 grows poorly even on DDO plates, suggesting that expressing this combination of plasmids results in poor growth in all conditions, rather than reduced level of interaction, underscoring why it is important to show these data. We removed the F678 samples, since the reviewer is correct that this mutant is not relevant. We have also included more information about the selection in the methods section. Lines 588-595. We used full-length Rabaptin-5 and have clarified this in the figure legend.

9. In Fig. S3, what are HA-HD-PTP-FL-WT and HA-HD-PTP-FL-LI/DD (what is "FL"?)? I assume the cells are transfected with GFP-RBPT5 and an HA-tagged HD-PTP (WT or the LI/DD). It would be clearer to state what the cells were transfected with at the top and then label the panels going down with GFP, HA, Rab5.
- We have defined FL as full-length, including the figure legend. We ensure that LI/DD is defined in the text. We have modified the figure panels as requested.

10. In Fig. 5F, how do lanes 1 and 2, and 4 and 5 differ? The legend does not explain, but it looks as though GFP alone has been used as a control.
- We have improved the figure labelling to make this clearer.

11. Fig. 6A and B. While I agree that in Fig. 6A less CC2-2 seems to be pulled down with HD-PTP-Bro1-CC with the CHMP4B-binding pocket mutated (lines 308-312), in the gel in Fig. 6B it looks as though the same is true for CC2-1. I see no band at all in the figure, though a band is clear for the pull down of CC2-1 with WT-HD-PTP. The graph suggests otherwise. Is the gel not a particularly good example here? I assume the graph uses averages of several experiments, so another gel should be substituted.
- We have replaced the figure panel, and have also included fuller statistical descriptions within the histogram to highlight where differences are not significant. We have toned down the text here somewhat, substituting "the modest binding of Rabaptin-5 CC2-1 to HD-PTP-Bro1-CC was unaffected by this mutation" to "was not significantly affected by this mutation". Lines 307-308.

12. Fig. 6C. What does the sh stand for in e.g. WT CHMP4B.sh?
- This unnecessary detail (sh actually refers to short; we had both long and short versions of the peptides) has been removed.

13. Fig. 6D. Do all these mutations affect endofin interaction? I think more than one sentence is needed to explain this figure (lines 328-329). Why do some mutations have more effect on Rabaptin-5 binding than others?
- We have made the text more explicit here, to explain the S pocket and describe the effects of the mutations on endofin binding, rather than simply refer to a previous paper. Lines 324-334.

© 2021. Published by The Company of Biologists under the terms of the Creative Commons Attribution License (https://creativecommons.org/licenses/by/4.0/).

Reviewer 3

a. The title is somewhat misleading to a reader not familiar with HDPTP. Since the work is about an increases in phosphorylation of Rabaptin upon HDPTP inhibition, a reader may think that HDPTP is the phosphatase. A mention of this in the abstract (saying HDPTP is probably not be the phosphatase) maybe better. Otherwise the authors would have to perform in-vitro assays to demonstrate phosphatase activity of HDPTP.

- We have included a sentence in the abstract indicating that HD-PTP is unlikely to be the direct phosphatase for Rabaptin-5. Lines 37-40.

b. Related to Fig 2: in the text, it is not mentioned that Alix knockdown does have an effect in MVB sorting. This should be mentioned explicitly, unless the authors are suggesting that Alix is not important for MVB sorting, which would be surprising to me. Fig. 2 does show punctate structures that are supposed to be un-degraded EGFR.

- We have stated explicitly that Alix depletion impairs EGFR degradation, and provided references to previous work. Lines 156-159.

c. HD-PTP binds Rabaptin at the CHMP4-binding site. The specificity of this interaction is provided by the “S-pocket” in HDPTP, which is not present in Alix. Some sequence/structural information about this pocket as an accompanying figure in Fig. 6 would be helpful for the readers, to demonstrate this interesting difference between HDPTP and Alix.

- We have included significantly more information in the text about the S pocket. Lines 324-334. We have also included information about the key residues in the S pocket in Figure 7D.

d. In the Discussion, line 345, I’d suggest replacing the word “control” with a less strong word. While I agree from the data that HDPTP binds to Rabaptin and is involved in facilitating endosomal maturation with MVB sorting, I’m not sure we can say that HDPTP controls endosome maturation.

- We have altered the wording accordingly. Line 348.

Second decision letter

MS ID#: JOCES/2021/259192

MS TITLE: His Domain Protein Tyrosine Phosphatase and Rabaptin-5 couple endo-lysosomal sorting of EGFR with endosomal maturation

AUTHORS: Gabrielle Parkinson, Peristera Roboti, Ling Zhang, Sandra Taylor, and Philip Woodman
ARTICLE TYPE: Research Article

I am happy to tell you that your manuscript has been accepted for publication in Journal of Cell Science, pending standard ethics checks.

© 2021. Published by The Company of Biologists under the terms of the Creative Commons Attribution License (https://creativecommons.org/licenses/by/4.0/).
